# Transcriptional profiling of human Vδ1 T cells reveals a pathogen-driven adaptive differentiation program

**DOI:** 10.1016/j.celrep.2022.110858

**Published:** 2022-05-24

**Authors:** Jack L. McMurray, Anouk von Borstel, Taher E. Taher, Eleni Syrimi, Graham S. Taylor, Maria Sharif, Jamie Rossjohn, Ester B.M. Remmerswaal, Frederike J. Bemelman, Felipe A. Vieira Braga, Xi Chen, Sarah A. Teichmann, Fiyaz Mohammed, Andrea A. Berry, Kirsten E. Lyke, Kim C. Williamson, Michael J.T. Stubbington, Martin S. Davey, Carrie R. Willcox, Benjamin E. Willcox

**Affiliations:** 1 Institute of Immunology and Immunotherapy, University of Birmingham, Birmingham B15 2TT, UK; 2 Infection and Immunity Program and Department of Biochemistry and Molecular Biology, Biomedicine Discovery Institute, Monash University, Clayton, VIC 3800, Australia; 3 Department of Haematology, Birmingham Children’s Hospital, Birmingham B4 6NH, UK; 4 Institute of Infection and Immunity, Cardiff University School of Medicine, Heath Park, Cardiff CF14 4XN, UK; 5 Australian Research Council Centre of Excellence in Advanced Molecular Imaging, Monash University, Clayton, VIC 3800, Australia; 6 Department of Experimental Immunology, Amsterdam Infection and Immunity Institute, Amsterdam UMC, University of Amsterdam, Amsterdam, the Netherlands; 7 Renal Transplant Unit, Division of Internal Medicine, Academic Medical Centre, Amsterdam UMC, University of Amsterdam, Amsterdam, the Netherlands; 8 Wellcome Sanger Institute, Cambridge, UK; 9 European Molecular Biology Laboratory, European Bioinformatics Institute (EMBL-EBI), Cambridge CB10 1SD, UK; 10 Center for Vaccine Development and Global Health, University of Maryland School of Medicine, Baltimore, MD, USA; 11 Department of Microbiology and Immunology, Uniformed Services University of the Health Sciences, Bethesda, MD, USA; 12 Present address: Amsterdam UMC, Meibergdreef 9, 1105 Amsterdam, the Netherlands; 13 Present address: Department of Biology, Southern University of Science and Technology of China, Shenzhen, China; 14 Present address: 103 Genomics Inc., 6230 Stoneridge Mall Road, Pleasanton, CA 94588, USA; 15 These authors contributed equally; 16 Lead contact

## Abstract

γδ T cells are generally considered innate-like lymphocytes, however, an ‘‘adaptive-like’’ γδ compartment has now emerged. To understand transcriptional regulation of adaptive γδ T cell immunobiology, we combined single-cell transcriptomics, T cell receptor (TCR)-clonotype assignment, ATAC-seq, and immunophenotyping. We show that adult Vδ1^+^ T cells segregate into TCF7^+^LEF1^+^Granzyme B^neg^ (T_naive_) or T-bet^+^Eomes^+^ BLIMP-1^+^Granzyme B^+^ (T_effector_) transcriptional subtypes, with clonotypically expanded TCRs detected exclusively in T_effector_ cells. Transcriptional reprogramming mirrors changes within CD8^+^ αβ T cells following antigen-specific maturation and involves chromatin remodeling, enhancing cytokine production and cytotoxicity. Consistent with this, *in vitro* TCR engagement induces comparable BLIMP-1, Eomes, and T-bet expression in naive Vδ1^+^ and CD8^+^ T cells. Finally, both human cytomegalovirus and *Plasmodium falciparum* infection *in vivo* drive adaptive Vδ1 T cell differentiation from T_naive_ to T_effector_ transcriptional status, alongside clonotypic expansion. Contrastingly, semi-invariant Vγ9^+^Vδ2^+^ T cells exhibit a distinct ‘‘innate-effector’’ transcriptional program established by early childhood. In summary, adaptive-like γδ subsets undergo a pathogen-driven differentiation process analogous to conventional CD8^+^ T cells.

## In brief

Using single-cell transcriptomics, TCR repertoire analysis, ATAC-seq, and immunophenotyping, McMurray et al. show naive Vδ1^+^ T cells can undergo transcriptional reprogramming to an effector state extremely similar to CD8 T_EMRA_ cells. Infections, including CMV and malaria, drive both clonotypic Vδ1^+^ T cell expansion and differentiation to this highly conserved effector program.

## Graphical Abstract

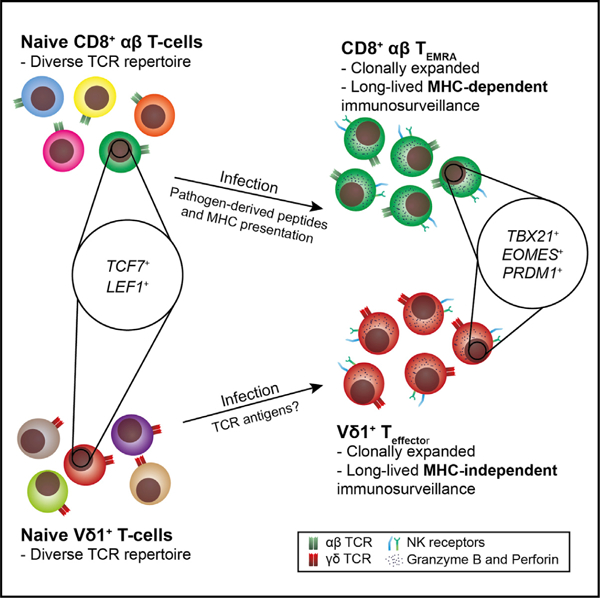

## INTRODUCTION

γδ T cells have been retained alongside αβ T cells and B cells for ~450 million years, with each compartment expressing distinct but related somatically recombined antigen receptors (Hayday, 2000; Vantourout and Hayday, 2013). Functioning in both anti-microbial and anti-tumor immunity (Silva-Santos et al., 2019; Vantourout and Hayday, 2013), γδ T cells display a distinct tissue distribution, can be enriched at epithelial surfaces, and employ unique recognition modes to sense target cells dysregulated by infection or transformation (Willcox and Willcox, 2019; Willcox et al., 2020). This poorly understood process involves a combination of γδ T cell receptor (TCR)-intrinsic but non-major histocompatibility complex (MHC)-restricted interactions, and TCR-extrinsic receptor/ligand interactions (Willcox and Willcox, 2019). The differentiation status of responding γδ T cells likely critically affects the outcome of such recognition processes.

How established lymphocyte differentiation paradigms apply to human γδ T cell function is unclear. While γδ T cells are often assumed to be ‘‘innate-like,’’ or ‘‘bridging innate and adaptive immunity,’’ increasing evidence suggests that parallel innate-like and adaptive paradigms operate in the human γδ T cell compartment, and apply to discrete γδ T cell subsets. Vγ9^+^Vδ2^+^ T cells prevalent in adult peripheral blood (PB) seem to adopt an innate-like immunobiology (Davey et al., 2018b; Willcox et al., 2018). They display a semi-invariant TCR repertoire from birth (Davey et al., 2018b), respond polyclonally to host and microbially derived phosphoantigens (Davey et al., 2018b), and display polyclonal recognition of butyrophilin superfamily (BTN)-2A1 via germline-encoded regions of the Vγ9^+^ TCR chain (Karunakaran et al., 2020; Rigau et al., 2020). Similarly, human intestinal γδ T cells include an intraepithelial lymphocyte (IEL) population polyclonally reactive to butyrophilin-like 3.8 heterodimer via germline-encoded regions of the Vγ4^+^ TCR chain (Melandri et al., 2018; Willcox et al., 2019), likely reflecting a distinct innate-like biology.

In contrast, PB Vδ1^+^ T cells adopt a more adaptive-like immunobiology (Davey et al., 2017; Ravens et al., 2017), as they display highly focused clonal expansion, including after viral (Farnault et al., 2013; Fujishima et al., 2007; Ravens et al., 2017) or parasitic (Rutishauser et al., 2020; von Borstel et al., 2021) infection, concomitant with apparent differentiation of select clones from a TCR-diverse T_naive_ pool to generate long-lived, TCR-focused T_effector_ populations (Davey et al., 2018a; Davey et al., 2017; von Borstel et al., 2021). Also, Vδ1^+^ T_naive_ and T_effector_ subsets bear immunophenotypic similarities to conventional adaptive CD8^+^ T_naive_ and T_EMRA_ populations, respectively (Davey et al., 2018a). Recently we defined a subset of Vγ9^neg^Vδ2^+^ T cells that shares critical adaptive-like hallmarks with PB Vδ1^+^ T cells (Davey et al., 2018b) and is linked to anti-viral (Davey et al., 2018b; Kaminski et al., 2020) and autoimmune (Bank et al., 1995; Hohlfeld et al., 1991; Pluschke et al., 1992) responses. The relevance of these observations likely extends to solid tissues. Hepatic γδ T cells included heavily expanded Vδ1^+^ and Vγ9^neg^Vδ2^+^ T_effector_ clonotypes, with some Vδ1^+^ clonotypes shared with PB, and Vδ1^+^ T_effector_ prevalence linked to viral exposure (Davey et al., 2018b; Hunter et al., 2018). Moreover, adaptive-like Vγ4^neg^Vδ1^+^ T cells were recently shown to outcompete endogenous γδ T cell populations during intestinal autoimmunity (Mayassi et al., 2019). In summary, a seemingly adaptive-like γδ immunobiology appears to drive potent T cell responses in several contexts, including viral and parasitic infections, and in autoimmunity.

While conventional αβ T cell differentiation is relatively well understood, the transcriptional basis of adaptive-like γδ T cell differentiation has not been addressed. CD8^+^ T cells transition from a TCR-diverse naive status through a series of differentiation states, via altered expression of a set of critical transcription factors (Fu et al., 2017; Henning et al., 2018; Kaech and Cui, 2012; Lazarevic et al., 2013; Vieira Braga et al., 2015). Initial antigen recognition and TCR signaling, culminating in clonal expansion, is considered critical in initiating this adaptive transition. Whether this model applies to adaptive-like γδ T cell populations is unclear. To date, transcriptional analyses of γδ T cells have stressed features of ‘‘innateness’’ shared between innate-like γδ subsets and unconventional αβ T cells, notably mucosal-associated innate T cells (MAITs) and invariant natural killer T cells (iNKTs) (Gutierrez-Arcelus et al., 2019; Koay et al., 2019; Pizzolato et al., 2019), but have not delineated differentiation processes fundamental to ‘‘adaptive-like’’ γδ T cell responses. To address this, we analyzed the transcriptional paradigms underpinning human γδ T cell function in both adaptive-like ( Vδ1^+^, Vγ9^neg^Vδ2^+^) and innate-like ( Vγ9^+^Vδ2^+^) subsets. We reveal striking similarities in transcriptional regulatory mechanisms between adaptive-like γδ T cell and CD8^+^ αβ T cell populations, including a tight link between clonotypic expansion and differentiation, and suggest they adopt a fundamentally different transcriptional control paradigm to innate-like Vγ9^+^Vδ2^+^ T cells.

## RESULTS

### Human PB Vδ1^+^ T cells exhibit two transcriptional profiles

**Figure 1.**
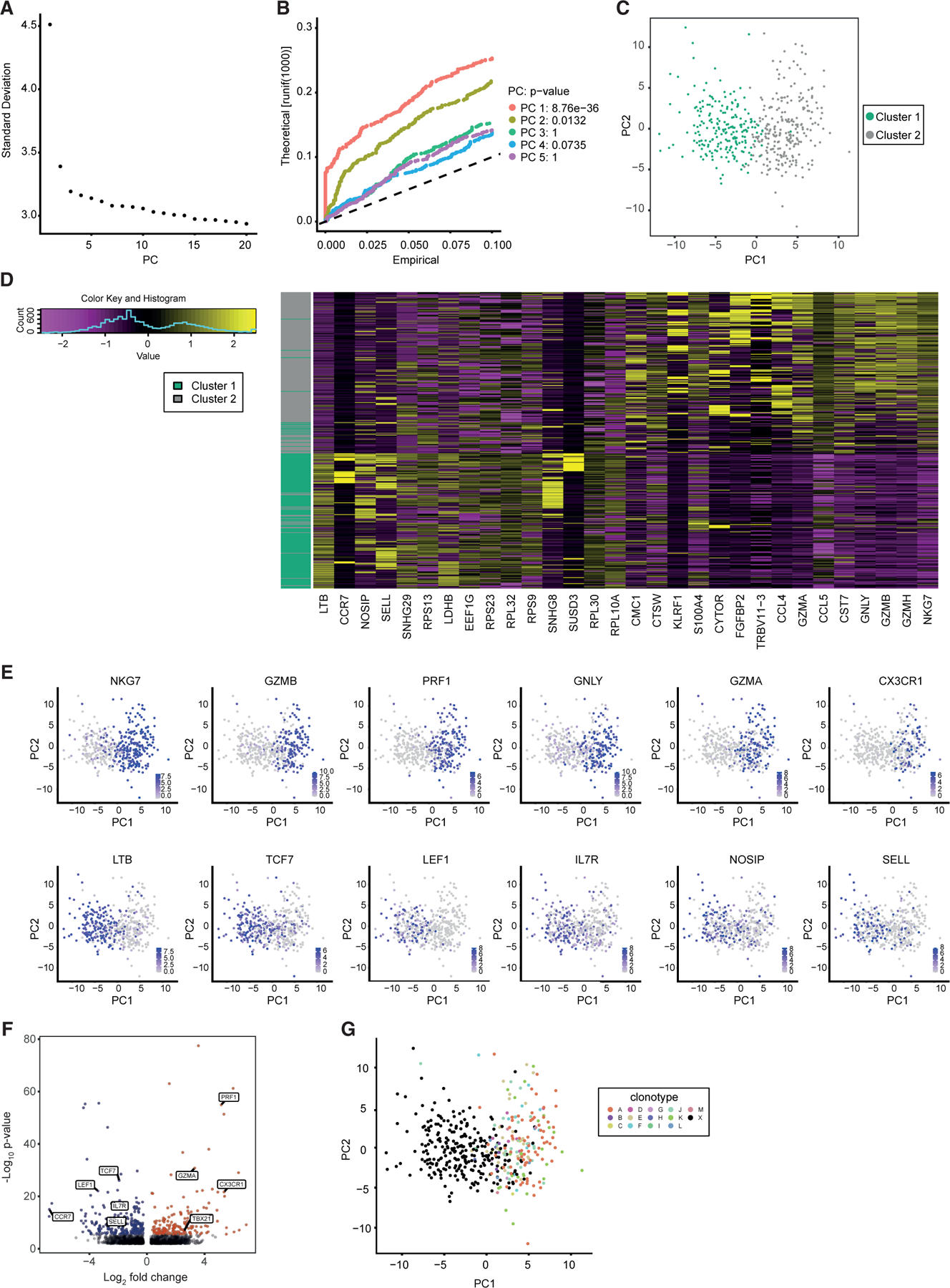
Clustering of single-cell transcriptomes reveals two distinct states of Vδ1^+^ cells reminiscent of effector and naive phenotypes (A and B) Principal-component analysis was performed on single-cell RNA sequencing data from 447 FACS-sorted Vδ1^+^ cells (CD27^hi^ versus CD27^lo/neg^) from three donors. (A) An elbow plot was constructed using the 20 dimensions. An ‘‘elbow’’ is observed at PC2. (B) A JackStraw plot was used to investigate the top 5 principal components (PCs). PC1 and PC2 were significant and used for visualization. (C) Shared nearest neighbor clustering was used to find clusters in the data. Two clusters were found and colored. (D) A heatmap was created using the top 30 genes contributing to the differences between cluster 1 and 2 (PC1). An even number of positive and negative factors are shown. These highlight genes previously shown to be associated with effector or naive status. (E) Expression of genes previously shown to be associated with either effector (top) or naive (bottom) status were projected onto the PCA plot. Purple indicates higher expression. (F) Differential gene expression between cluster 1 (blue) and cluster 2 (red) cells revealed 368 differentially expressed genes. Only those that had a LogFC greater than 0.25 and an adjusted p value of less than 0.05 were colored (Bonferroni corrected). Notable genes previously associated with effector or naive status are labeled. (G) TCR clonality analysis. Thirteen expanded clonotypes were identified by the analysis (clones A–M), and projected onto the PCA taken from (B). ‘‘X’’ represents cells with a unique TCR sequence. Cells bearing expanded clonotypes fall within CD27^lo/neg^ sorted cells and almost exclusively within the ‘‘T_effector_’’ cluster (cluster 2).

To understand adaptive-like γδ T cell differentiation, we performed single-cell transcriptomic analyses of human Vδ1^+^ T cells. Vδ1^+^ T cells include distinct phenotypically naive (CD27^hi^) and effector-like (CD27^lo/neg^) sub-compartments (Davey et al., 2017, 2018a). A total of 447 single Vδ1^+^ T cells were sorted from 3 individuals with cell surface staining for CD27 and CD45RA; roughly equal numbers of CD27^hi^ and CD27^lo/neg^ cells were sorted (Figures S1A and S1B), with acceptable quality control statistics (transcripts per cell, genes per cell, fraction of mitochondrial reads per cell) (Figure S1C). Principal-component dimension reduction analysis and subsequent unsupervised clustering of Vδ1^+^ T cell transcriptomes identified two clusters of cells (Figures 1A–1C).

Differential gene expression comparisons between cluster 1 and cluster 2 Vδ1^+^ T cells revealed 368 differentially expressed genes (Table S1). Cluster 1 Vδ1^+^ T cells exhibited enhanced expression of *SELL*, *IL7R*, and *CCR7* and low expression of effector-related genes, including *CX3CR1*, *GZMA*, and *PRF1* (Figures 1D–1F), whereas cluster 2 Vδ1^+^ T cells had an inverse expression pattern. This indicated close concordance with cell surface Vδ1^+^ T_naive_ and T_efffector_ (cluster 1 and cluster 2, respectively) phenotypes we previously defined and noted were similar to CD8^+^ T_naive_ and T_EMRA_ cells, respectively (Davey et al., 2017, 2018a). Indexing of individual transcriptomes indicated a 91.1% concordance with flow phenotype (Figure S1D), whereby cluster 1 Vδ1^+^ T cells were predominantly CD27^hi^ and cluster 2 Vδ1^+^ T cells CD27^lo^, confirming Vδ1^+^ T_naive_ and T_effector_ status, respectively (Figures S1E–S1H). While cluster 1 and 2 Vδ1^+^ T cell transcriptomes (referred to as Vδ1^+^ T_naive_ and T_effector_ programs) differentially expressed several key transcription factors, including *TCF7*, *LEF1*, and *TBX21* (Figure 1F), the challenges of detecting low expression genes using single-cell transcriptomics suggested that this was an incomplete list.

We next probed the link between Vδ1^+^ clonotypic expansion and possession of a T_naive_/T_effector_ program. We extended the TraCeR algorithm (Stubbington et al., 2016) to reconstruct human γ and δ TCR sequences from standard single-cell transcriptomes. This leveraged approaches previously applied to αβ TCR sequence extraction (Stubbington et al., 2016), although the γ and δ complementarity determining region 3 length parameters were informed by bulk TCR sequencing on human γδ T cells (Davey et al., 2017, 2018b; Hunter et al., 2018). We reconstructed both γ and δ TCR sequences from ~70% of cells, a similar success rate to αβ T cells (Stubbington et al., 2016). Whereas γδ TCR clonotypes assigned to the T_naive_ cellular cluster were overwhelmingly distinct ‘‘singlets’’ (95.6%), expanded clonotypes (those observed in more than one cell) were restricted almost entirely (95.3%) to Vδ1^+^ cells bearing a T_effector_ transcriptional program (cluster 2) (Figure 1G). Compelling statistical significance (p < 2.2e–16, Pearson’s chi-square test, Yates continuity correction) was evident in three associations: cluster status (1 or 2) with sorted phenotype (CD27^hi^ or CD27^lo/neg^); clonality status (singlet or expanded clonotype) with cluster status (1 or 2); between clonality status (singlet or expanded clonotype) and sort phenotype (CD27^hi^ or CD27^lo/neg^) (Figures S1E–S1H). Finally, TCR sequence analysis enabled us to probe transcriptional differences between distinct expanded clonotypes. While few interclonal differences were observed, two observations validated the approach. Firstly, interclonal discrepancies in Vγ chain expression were apparent and, secondly, expression of *XIST*, an X-linked transcript involved in X chromosome inactivation in females, was restricted to the female donor’s major clone (Figures S1I and S1J; Table S2).

These results suggested that distinct naive and effector transcriptional programs underpin Vδ1^+^ T cell differentiation, that these two states can be accurately delineated by CD27 status, and established that effector status was intrinsically linked to clonotypic expansion at a single-cell level.

### Analogous transcriptional reprogramming in CD8^+^ and Vδ1^+^ T cells

**Figure 2.**
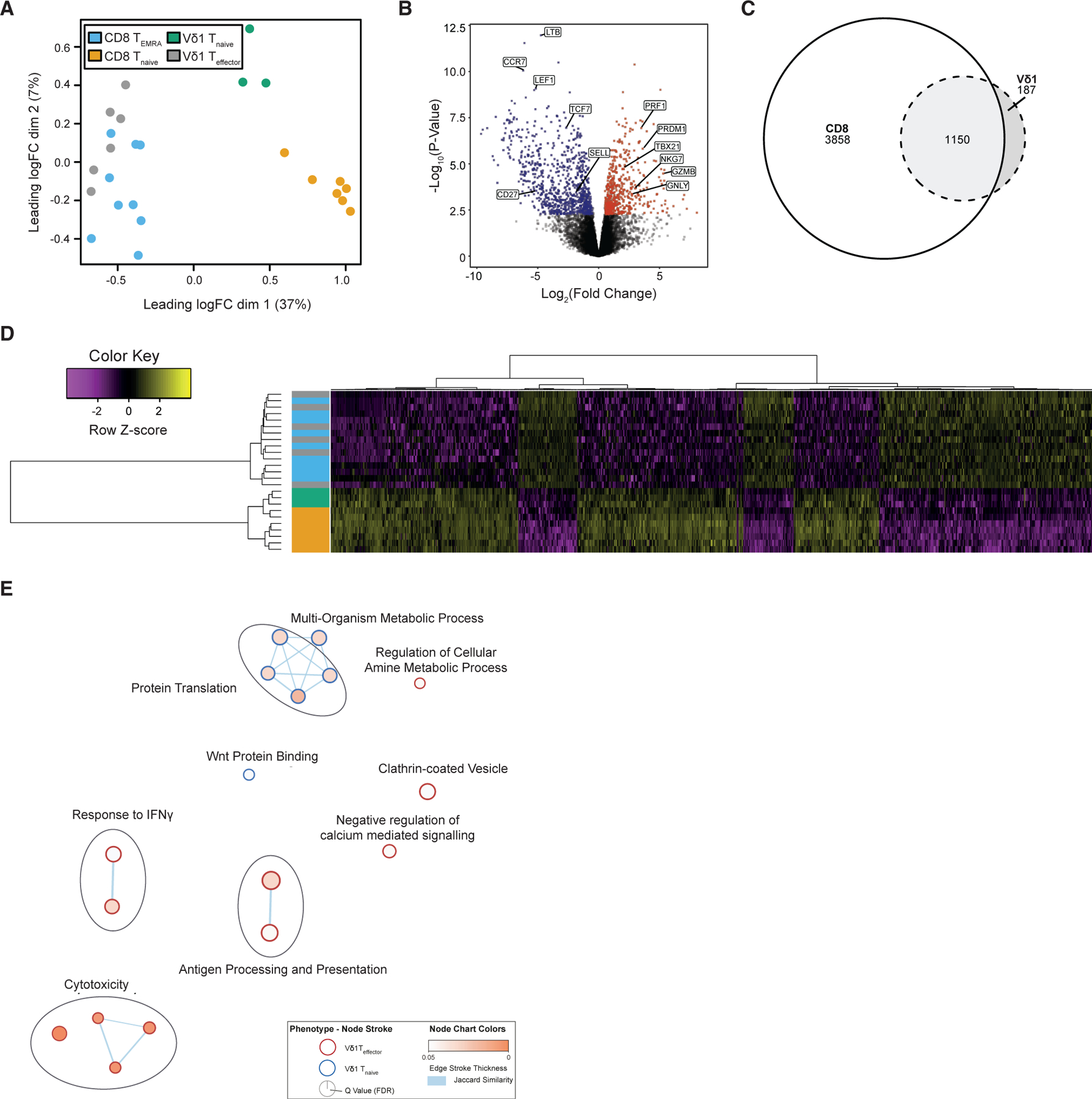
Bulk RNA sequencing on sorted Vδ1 populations reveals transcriptomic similarity between Vδ1^+^ and CD8^+^ populations (A) Multidimensional scaling plot using 12,627 filtered genes revealed close transcriptomic similarity between bulk sorted Vδ1^+^ T_effector_ and CD8^+^ T_EMRA_. (B) Differential gene expression between Vδ1^+^ T_effector_ (red) and Vδ1^+^ T_naive_ (blue) revealed 1,337 differentially expressed transcripts. Among these were genes related to effector or naive status. (C) Differential gene expression between Vδ1^+^ and CD8^+^ populations revealed a core of 1,150 differentially expressed transcripts shared between comparisons. (D) Hierarchically clustered heatmap of the core 1,150 transcripts differentially expressed in both Vδ1^+^ and CD8^+^ comparisons highlighted similarity between Vδ1^+^ T_effector_ and CD8^+^ T_EMRA_ cells and between Vδ1^+^ T_naive_ and CD8^+^ T_naive_ cells. Colors for the cell populations are taken from (A). Genes are plotted row-wise and scaled to represent *Z* scores across samples. (E) Gene set enrichment analysis using the GO and KEGG databases revealed enrichment of 11 pathways associated with effector function in Vδ1^+^ T_effector_ cells. In contrast, 6 pathways were associated with Vδ1^+^ T_naive_ status. Genes differentially expressed between Vδ1^+^ populations were used as input. Cytoscape was used to produce the enrichment map, where node size indicates the gene set size, edge thickness represents Jaccard similarity scores between gene sets, and node color is based on FDR value. Only gene sets with an FDR below 0.05 are shown.

To gain a deeper knowledge of the Vδ1^+^ T_naive_ and T_effector_ transcriptional programs and how they compare with CD8^+^ differentiation states with a similar cell surface phenotype, we performed bulk RNA-seq on Vδ1^+^ T_naive_ (CD27^hi^) and T_effector_ (CD27^lo^) populations and both CD8^+^ T_naive_ and T_EMRA_ populations, sorted from human PB mononuclear cells from healthy donors or buffy packs (see STAR Methods). Using all filtered genes, multidimensional scaling revealed strong similarity between CD8^+^ T_EMRA_ and Vδ1^+^ T_effector_ populations (Figure 2A), both of which were clearly separated from CD8^+^ T_naive_ and Vδ1^+^ T_naive_ populations in dimension 1. While Vδ1^+^ T_naive_ and CD8^+^ T_naive_ cells were broadly similar transcriptionally, they exhibited some differences, particularly in dimension 2; however, this made a minor contribution to the observed variance (7%). In contrast, Vδ1^+^ T_naive_ and CD8^+^ T_naive_ cells were more concordant in dimension 1, which made a larger contribution (37%).

These data are in accordance with, but extend, our previous phenotypic data on adaptive-like Vδ1^+^ T cells (Davey et al., 2017, 2018a). Differential gene expression analysis between Vδ1^+^ T_naive_/T_effector_ populations revealed 1,337 differentially expressed transcripts (Figure 2B), substantially more than revealed by single-cell analysis.

We next compared genes differentially expressed in both Vδ1^+^ and CD8^+^ subpopulations. Interestingly, 86% of all genes differentially expressed between Vδ1^+^ T_naive_/T_effector_ populations were also differentially expressed between CD8^+^ T_naive_/T_EMRA_ (Figure 2C). Within this conserved set of 1,150 differentially expressed genes were those canonically related with effector (*GNLY*, *GZMA*/*B*/*H*, and *PRF1*) and naive (*CCR7*, *LEF1*) status (Figure 2B). Genes downregulated in Vδ1^+^ T_effector_ cells contributed more strongly than upregulated genes to this core gene list (Figure S2A). Heatmap analysis, using hierarchical clustering, on this 1,150-gene list segregated Vδ1^+^ T_effector_ cells with CD8^+^ T_EMRA_ cells, and the Vδ1^+^ T_naive_ subset alongside CD8^+^ T_naive_ cells, highlighting clear concordance of gene expression within each subgroup (Figure 2D). Interestingly, Vδ1^+^ T_effector_ populations were not clustered together as a group, but instead clustered among CD8^+^ T_EMRA_ populations, emphasizing close transcriptional concordance (Figure 2D). In contrast, T_naive_ subsets clustered separately by compartment ( Vδ1^+^ and CD8^+^ T cells) (Figure 2D), consistent with greater transcriptional differences between naive Vδ1^+^ and CD8^+^ T cells (Figure 2A).

Gene set enrichment analysis highlighted 11 pathways significantly upregulated in Vδ1^+^ T_effector_ versus Vδ1^+^ T_naive_ subsets (Figures 2E; Table S3). Chief among these were effector/cytotoxicity, antigen processing/presentation, and IFN-γ-responsive genes, mirroring changes in CD8^+^ T_EMRA_ versus T_naive_ compartments (Figures S2B; Table S3). Also, analysis of bespoke gene sets linked to cytotoxic molecules (Figure S3A), cytokine and chemokine receptors (Figure S3B), adhesion molecules (Figure S3C), and TCR/co-stimulatory molecules (Figure S3D) underlined close concordance between Vδ1^+^ T_effector_ and CD8^+^ T_EMRA_ cells. Conversely, a minority of processes were upregulated on Vδ1^+^ T_naive_ cells relative to Vδ1^+^ T_effector_ cells, including protein translation and metabolism (Figure 2E); these were also upregulated on CD8^+^ T_naive_ cells versus CD8^+^ T_EMRA_ cells (Figure S2B). Of note, several activating co-stimulatory molecules (e.g., *CD28*, *CD27*, and *JAML*) displayed higher expression on both Vδ1^+^ T_naive_ and CD8^+^ T_naive_ cells relative to Vδ1^+^ T_effector_ and CD8+ T_EMRA_ subsets, whereas several inhibitory co-signaling molecules (e.g., *PDCD1*, *TIGIT*, and *LAG3*) were increased on T_effector/EMRA_ cells (Figure S3D). Furthermore, numerous NKRs, including *KLRK1* (NKG2D), *NCR1* (NKp46), and *KLRF1* (NKp80), were upregulated in Vδ1^+^ T_effector_ and CD8^+^ T_EMRA_ cells relative to T_naive_ subsets (Figure S3E). Vδ1^+^ and CD8^+^ T_effector_ subset concordance was close, with differential gene expression analysis revealing just 20 transcripts (Table S1). Of these, seven were TCR chain-specific genes, and two, *CD8A* and *CD8B*, known to be expressed differentially in CD8^+^ αβ versus γδ T cells, leaving 11 genes. Several of these (*CD5*, *THEMIS*, and *SIT1*) have established roles regulating TCR signaling thresholds in response to peptide MHC (Fu et al., 2009; Simeoni et al., 2005; Voisinne et al., 2018), and were preferentially expressed in CD8^+^ T cells. *ITGAD* was upregulated in Vδ1^+^ T_effector_ cells, consistent with previous findings (Siegers et al., 2017). Notably, the Vδ1^+^ T_naive_ compartment displayed marginally higher transcription of several NKR genes than CD8^+^ T_naive_ cells, with some specific NKRs (NKG2A/C/E (*KLRC1/3*), and *FCRL3*) displaying particularly high relative expression in the Vδ1^+^ T_naive_ compartment (Figure S3E).

### A shared set of TCR-linked transcription factors underpin Vδ1^+^ and CD8^+^ T cell differentiation

**Figure 3.**
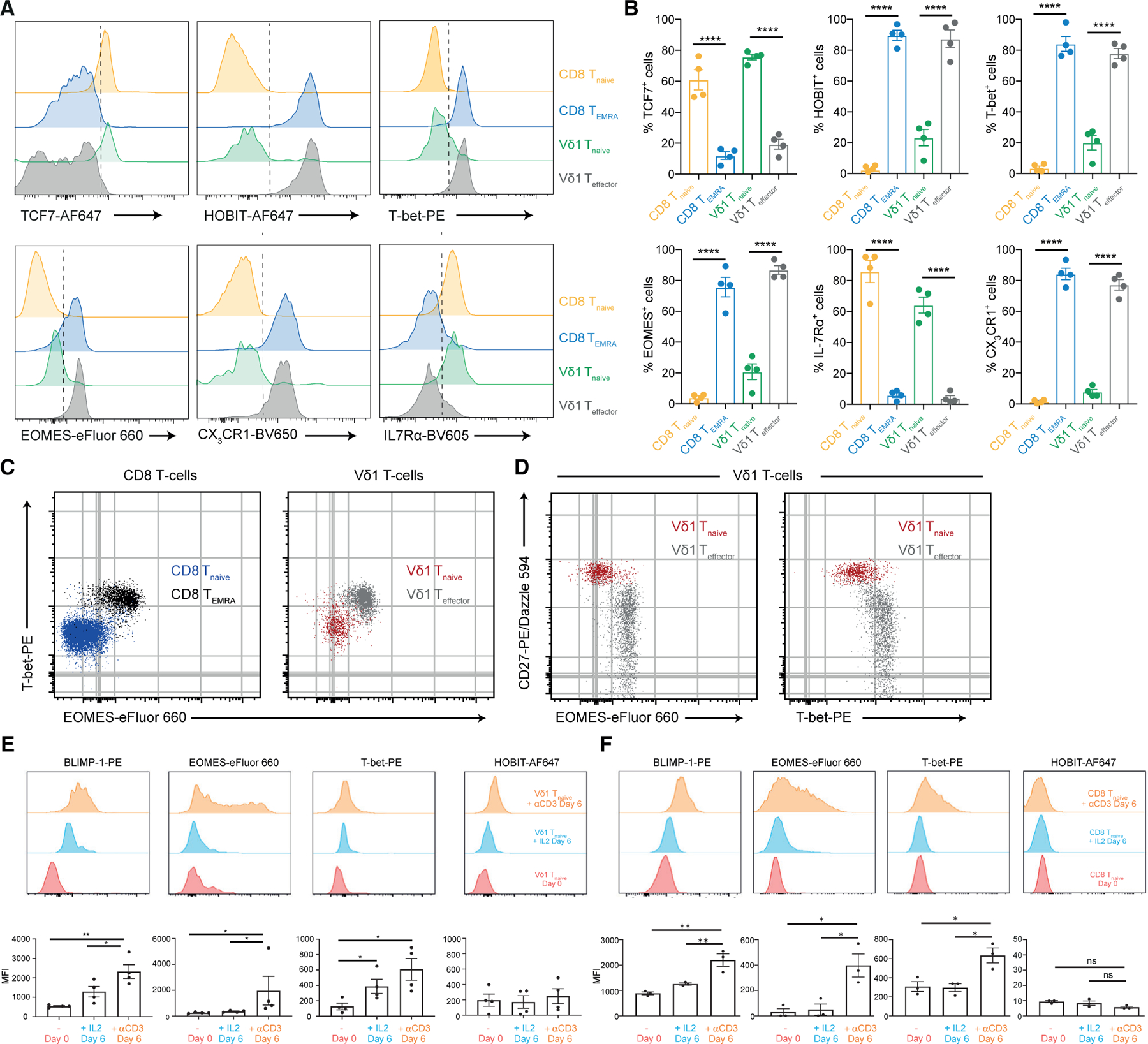
Transcription factors key to CD8^+^ differentiation demonstrate analogous and TCR-dependent expression in Vδ1^+^ subsets (A) Flow cytometry staining of transcription factors and homing receptors key to effector/naive status highlight similarities between Vδ1^+^ T_effector_ and CD8^+^ T_EMRA_, and Vδ1^+^ T_naive_ and CD8^+^ T_naive_ populations. Representative of four donors. (B) Percentage of each naive and effector marker in each total cell population (CD8^+^ T_naive_, CD8^+^ T_EMRA_, Vδ1^+^ T_naive_, Vδ1^+^ T_effector_). Graphs show mean ± SEM from four donors. (C and D) Overlaid flow cytometry dot plots of the transcription factors Eomes and T-bet in total Vδ1^+^ and CD8^+^ populations (C) and the overlap of each transcription factor with CD27 (D). Representative of four donors. Data were analyzed by one-way ANOVA with Tukey’s multiple comparisons (****p < 0.0001). (E and F) TCR stimulation of enriched T_naive_ cells leads to expression of T_effector_ transcription factors. Flow cytometric analysis of BLIMP-1, Eomes, T-bet, and HOBIT in Vδ1^+^ T_naive_ (E) and CD8^+^ T_naive_ (F) on day 0 (unstimulated) and day 6 of stimulation with either plate-bound anti-CD3 antibodies or IL-2. The top panel shows representative histograms for flow cytometric analysis, whereas the lower panel shows bar charts representing data from four donors. Data were analyzed by one-way ANOVA with Tukey’s multiple comparisons (*p < 0.05, **p < 0.01). Only significant comparisons are shown.

Transcriptional analyses also indicated differential expression of a core set of transcription factors delineated CD8^+^/Vδ1^+^ T_naive_ versus CD8^+^ T_EMRA_/Vδ1^+^ T_effector_ subsets. *TCF7*, *LEF1*, and *Myc* were enriched in T_naive_ subsets, whereas *TBX21* (T-bet), and *PRDM1* (BLIMP-1) were enriched in Vδ1^+^ T_effector_ cells and CD8^+^ T_EMRA_ cells (Figures 1E and 2B). Overall, 122 transcription factors were significantly differentially expressed between Vδ1^+^ T_naive_ and T_effector_ subsets (Table S4). Moreover, hierarchical clustering based on these transcription factors separated Vδ1^+^ T_naive_ with CD8^+^ T_naive_ cells as distinct clusters, whereas Vδ1^+^ T_effector_ cell clustering was interspersed with that of CD8^+^ T_EMRA_ cell populations (Figure S3F). Flow cytometry confirmed differential transcription factor expression at a protein level, establishing equivalently high TCF7 and IL-7Rα expression in Vδ1^+^ and CD8^+^ T_naive_ cells and absence in T_effector_ cells, whereas T-bet, Eomes, and BLIMP-1 were strikingly and equivalently upregulated in Vδ1^+^ T_effector_ and CD8^+^ T_EMRA_ cells relative to T_naive_ subsets (Figures 3A and 3B). Also, HOBIT expression was higher in Vδ1^+^ T_effector_ and CD8^+^ T_EMRA_ cells than T_naive_ cells (Figures 3A and 3B). T-bet and Eomes expression correlated with lower CD27 expression (Figures 3C and 3D). These changes correlated with established differentiation markers, as confirmed by assessment of IL7Rα (T_naive_) and CX3CR1 (Davey et al., 2017) (T_effector_/T_EMRA_) expression (Figures 3A and 3B). Of note, despite transcriptional differences in CXCR3 between Vδ1^+^ T_naive_ and CD8^+^ T_naive_ subsets, both expressed CXCR3 protein, whereas CXCR3 expression was lower on Vδ1^+^ T_effector_ and CD8^+^ T_EMRA_ cells (Figures S4A–S4C).

To address how Vδ1^+^ T_naive_ to T_effector_ transition affected chromatin structure, we carried out assay for transposase-accessible chromatin using sequencing (ATAC-seq) on purified Vδ1^+^ and CD8^+^ subpopulations (Figure S4D; Table S5). For a defined subset of loci, clear and analogous differences in chromatin accessibility were observed between CD8^+^/Vδ1^+^ T_naive_ populations and CD8^+^ T_EMRA_/Vδ1^+^ T_effector_ subsets (Figure S4D). These included *CD27*, which was more active in T_naive_ Vδ1^+^/CD8^+^ T cells than in T_EMRA_/Vδ1^+^ T_effector_ subsets. Conversely, *PRDM1* (BLIMP-1) and *EOMES* and *CX3CR1* all showed an inverse pattern of accessibility. In contrast, numerous loci displayed unaltered chromatin accessibility between CD8^+^/Vδ1^+^ T_naive_ populations and CD8^+^ T_EMRA_/Vδ1^+^ T_effector_ subsets, including *CD3E*, *B2M*, and *RPL13A* (Figure S4E). These results confirm that Vδ1^+^ T_naive_ and T_effector_ subsets represent two distinct differentiation states.

In CD8^+^ T cells, downregulation of TCF7/LEF1 and upregulation of Eomes, T-bet, and BLIMP-1 are associated with exposure of T_naive_ subsets to antigen-induced TCR signaling (Fu et al., 2017; Kaech and Cui, 2012; Lazarevic et al., 2013; Willinger et al., 2006). Consistent with this also operating in Vδ1^+^ T cells, Vδ1^+^ clonal expansion correlates with a transition from T_naive_ to T_effector_ status. To assess whether TCR signaling also induced similar changes in adaptive γδ T cells, we stimulated purified Vδ1^+^ T cells with plate-bound CD3 antibodies and assessed changes in transcription factor expression and phenotype in the Vδ1^+^ CD27^hi^ (i.e., T_naive_) subset (Figure 3E). Exposure of Vδ1^+^ T cells to CD3 stimulation induced BLIMP-1, Eomes, and T-bet expression in CD27^hi^ Vδ1^+^ T cells. TCR-induced expression of BLIMP-1 was previously shown for CD4^+^ T cells (Martins et al., 2006); however, here we show this extends to Vδ1^+^ and also CD8^+^ T cells (Figure 3F). While IL-2 (Figures 3E and 3F) and anti-CD28 (Figures S4F and S4G) appeared to amplify transcription factor expression, TCR signaling induced by anti-CD3 had the most profound effect on BLIMP-1, Eomes, and T-bet upregulation. This suggested that TCR signaling in Vδ1^+^ T_naive_ cells can initiate transition from T_naive_ to T_effector_ status, mirroring CD8^+^ T cell differentiation.

### The Vδ1^+^ T_effector_ program permits rapid cytokine production kinetics

**Figure 4.**
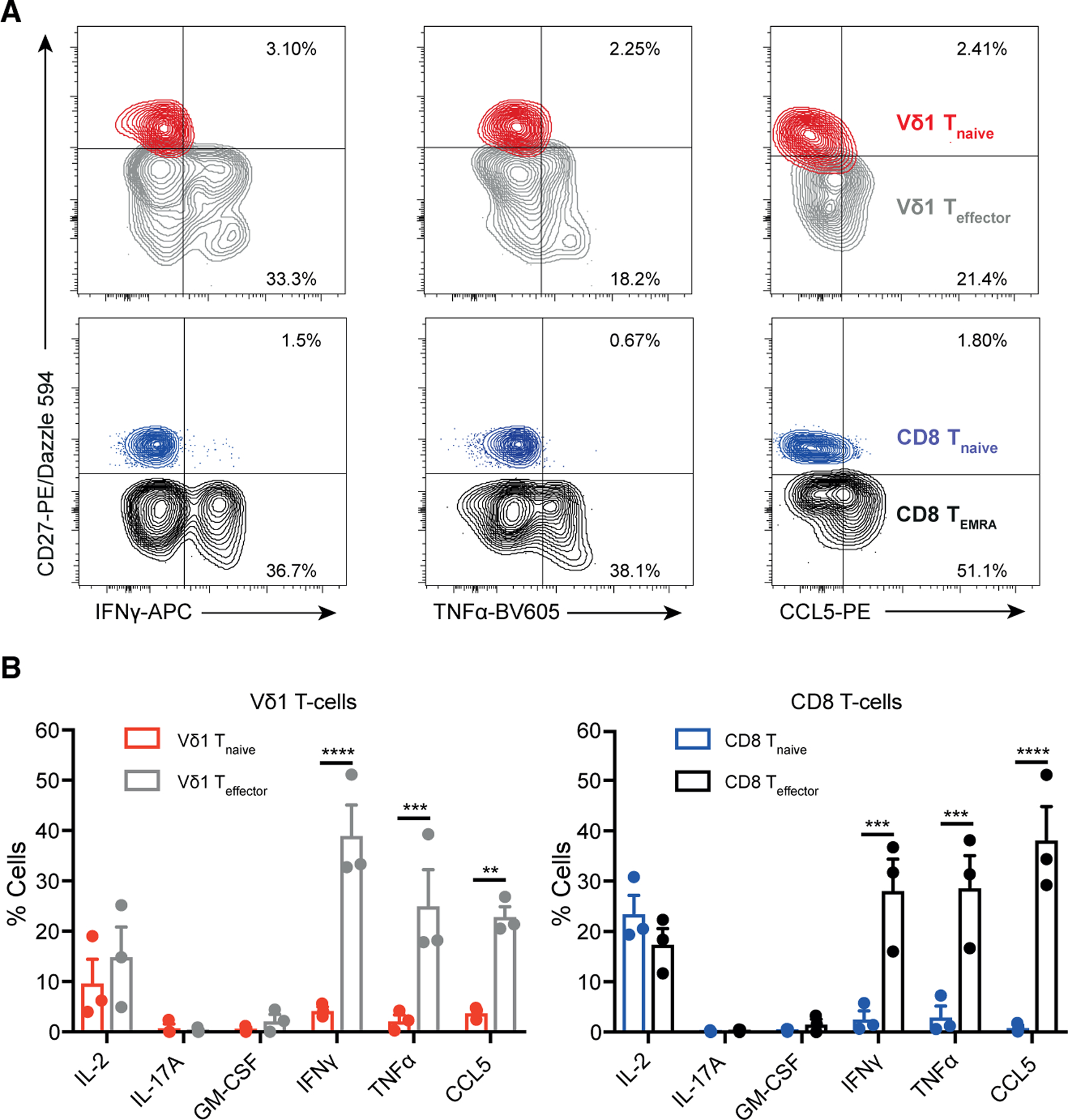
Vδ1^+^ T_effector_ and CD8^+^ T_EMRA_ populations display similar cytokine production profiles (A) Cytokine production by Vδ1^+^ T_effector_ and CD8^+^ T_EMRA_ and T_naive_ populations after 4 h of PMA/ionomycin stimulation. Overlaid contour flow cytometry plots of gated populations of Vδ1^+^ (top) and CD8^+^ (bottom). (B) Graphs highlight mean ± SEM and represent three donors. Data were analyzed by two-way ANOVA with Sidak’s multiple comparisons (*p < 0.05, **p < 0.01, ****p < 0.0001). Only significant comparisons are shown.

Production of inflammatory cytokines and enhanced cytotoxicity are hallmarks of γδ T cell responses. While cytotoxic pathways were upregulated at both the transcriptional and protein level in resting Vδ1^+^ T_effector_ cells, cytokines were not differentially transcribed in Vδ1^+^ T_effector_ relative to T_naive_ cells. Yet, ATAC-seq indicated substantially greater chromatin accessibility at the *IFN*γ locus in CD8^+^ T_EMRA_ than CD8^+^ T_naive_ cells. A similar pattern was observed in Vδ1^+^ subsets, albeit with a lower fold difference due to greater accessibility in Vδ1^+^ T_naive_ than CD8^+^ T_naive_ cells. This indicated the potential for enhanced/expedited IFN-γ production in T_effector_/T_EMRA_ subsets (Figure S4H). While similarly low ATAC-seq reads mapped to the TNF-α locus in T_naive_ and T_effector_/T_EMRA_ subsets, our analyses were performed on resting cells, and further signals may be needed to affect chromatin accessibility at this locus. Also, differential transcription factor expression in the T_naive_ versus T_effector_/T_EMRA_ states may influence cytokine production, as suggested for TCF7-mediated repression of IFN-γ expression in CD4 T cells (Maier et al., 2011). To test if their distinct chromatin and transcriptional profiles affected cytokine production, we stimulated both Vδ1^+^ T_naive_ and T_effector_ subsets and assessed production of IL-2, IL-17A, GM-CSF, IFN-γ, TNF-α, and CCL5. Vδ1^+^ T_effector_ cells displayed dramatically amplified and expedited production of IFN-γ, TNF-α, and CCL5 relative to Vδ1^+^ T_naive_ cells (but not IL-2, IL-17A, or GM-CSF) (Figures 4A, 4B, and S4I) in a manner highly analogous to CD8^+^ T_EMRA_ versus T_naive_ subsets. This suggests that Vδ1^+^ T_naive_ to T_effector_ transition, as for CD8^+^ T cells, results in transcriptional reprogramming and chromatin remodeling, enabling increased cytotoxicity and cytokine production, allowing amplified and expedited effector responses upon subsequent stimulation.

### Vγ9^+^Vδ2^+^ T cells display a distinct innate-like effector transcriptome from early life

**Figure 5.**
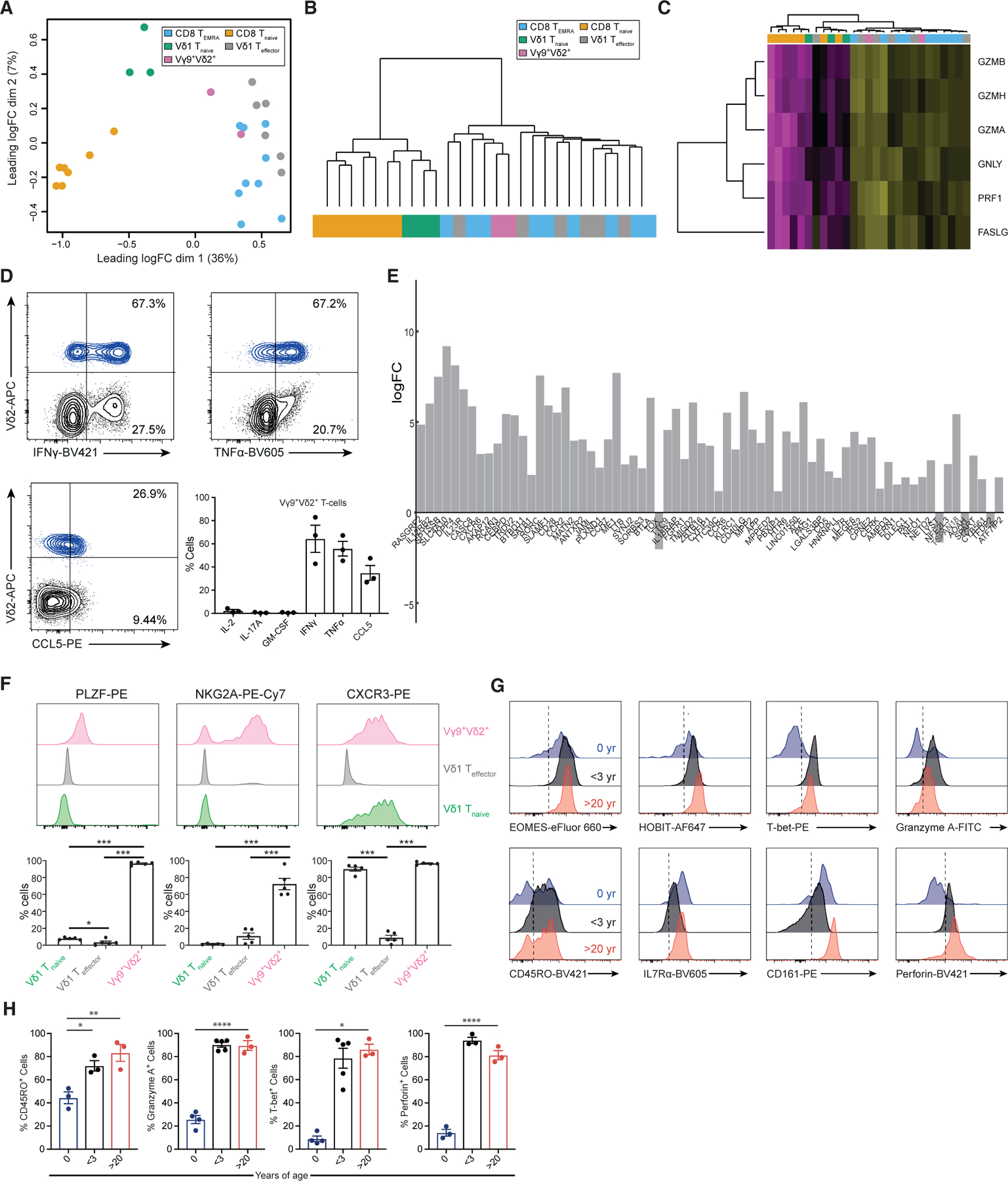
Transcriptomic analysis of Vδ2^+^ cells reveals an effector-like program (A) Multidimensional scaling plot on 12,627 filtered genes placed Vγ9^+^Vδ2^+^ T cells closer to Vδ1^+^ T_effector_ and CD8^+^ T_EMRA_ than to T_naive_ cells, highlighting increased transcriptional similarity of Vγ9^+^Vδ2 to effector populations. (B) Using the previously defined effector-naive gene list based on shared differentially expressed transcripts (1,150-gene list) between Vδ1^+^ and CD8^+^ populations, Vγ9^+^Vδ2^+^ cells were hierarchically clustered with the other effector populations. The dendrogram shown places Vδ2^+^ cells on the effector ‘‘arm,’’ suggesting more similar expression of these genes. Full heatmap in Figure S4A. (C) Vγ9^+^Vδ2^+^ cells segregated with the effector compartment based on a bespoke gene set of cytotoxic markers and were hierarchically clustered. Colors for the cell populations are taken from (A). Genes are plotted row-wise and scaled to represent *Z* scores across samples. (D) Cytokine production by Vγ9^+^Vδ2^+^ cells in total gated CD3^+^ T cells after 4 h PMA/ionomycin treatment. Flow plots are representative of three donors. Graphs depict mean ± SEM of an extended set of cytokine protein expression by intracellular flow cytometry. (E) Differential gene expression between Vγ9^+^Vδ2^+^ and Vδ1^+^ T_effector_ cells revealed close transcriptomic similarity, with 69 genes significantly differentially expressed (p < 0.05). Transcripts were ordered according to p value, with the most significant being *RASGRF2*, *IL12RB2*, and *SPTSSB*. A positive log fold change indicates an enrichment in Vγ9^+^Vδ2^+^ cells. (F) Vγ9^+^Vδ2^+^ T cells exhibit a unique innate-like effector program. Prevalence of γδ T cell subsets expressing PLZF, NKG2A, and CXCR3 was determined using flow cytometry. The top panel shows representative histograms for flow cytometric analysis; the lower panel shows bar charts representing data from five donors. Data were compared with one-way ANOVA with Tukey’s multiple comparisons (*p < 0.05, **p < 0.01). Only significant comparisons are shown. (G) Comparison of Vγ9^+^Vδ2^+^ T cell phenotype in cord blood, from unrelated children under 3 years of age, and from adult donors over 20 years of age. (H) Quantitation of MFI levels from (G), three samples of cord blood, five samples from children under the age of 3 years, and from three adult donors. Data were analyzed by two-way ANOVA with Sidak’s multiple comparisons (*p < 0.05, **p < 0.01). Only significant comparisons are shown.

RNA-seq data from Vγ9^+^Vδ2^+^ T cells sorted from healthy adults indicated an effector rather than naive transcriptional program. Multidimensional scaling (Figure 5A) positioned Vγ9^+^Vδ2^+^ cells closer to Vδ1^+^/CD8^+^ T_effector_ than T_naive_ populations. Unsupervised hierarchical clustering based on the 1,150 genes differentially expressed between T_naive_ and T_effector_ subsets also clustered Vγ9^+^ Vδ2^+^ cells with effector Vδ1^+^/CD8^+^ subsets (Figures 5B and S5A). Similar clustering approaches highlighted Vγ9^+^Vδ2^+^ T cell alignment with Vδ1^+^ T_effector_/CD8^+^ T_EMRA_ populations based on bespoke lists of cytotoxicity markers and NK receptors (Figures 5C and S5B). Moreover, Vγ9^+^Vδ2^+^ T cells were at least as potent producers of IFN-γ, TNF-α, and CCL5 upon anti-CD3 stimulation as Vδ1^+^ T_effector_ cells (Figures 4B and 5D).

Despite such similarities, several genes were enriched in Vγ9^+^Vδ2^+^ relative to Vδ1^+^ T_effector_ cells (Figure 5E; Table S1), including transcription factors (*CEBP*δ, *SATB1*, and *ROR*γ*t*), NKRs (*KLRK1* [NKG2A]), co-stimulatory molecules (*JAML*), cytokine receptors (*IL23R*), sphingolipid metabolism genes (*SPTSSB* and *KDSR*), and an alternative splicing gene (*HNRNPLL*), which is responsible for generation of the CD45RO isoform (Oberdoerffer et al., 2008) expressed by Vδ2^+^ T cells (Parker et al., 1990), but not generally by Vδ1^+^ T_effector_ or CD8^+^ T_EMRA_ cells. Vγ9^+^ Vδ2^+^ T cells also had a distinct overall profile of chemokine/cytokine receptors (Figure S5C) and transcription factors (Figure S5D) compared with Vδ1^+^ T_effector_/CD8^+^ T_EMRA_ subsets. While they shared expression of *TBX21*, *EOMES*, *ZEB2*, and *PRDM1* with these effectors, like T_naive_ subsets they were enriched for *Myc*, and also for both *ZBTB16* (PLZF, also expressed in iNKT and MAIT cells) and *CEBPD* (C/EBPδ), which is also expressed by MAIT cells and supports CCR6 expression (Lee et al., 2018) on Vγ9^+^Vδ2^+^ T cells (Figure S5C). Higher transcription of RORγt and IL-23R in Vγ9^+^Vδ2^+^ than in Vδ1^+^ T_effector_ cells is consistent with the suggestion that adult Vγ9^+^Vδ2^+^ T cells may retain some IL-17-producing cells (Tan et al., 2020). Also, flow cytometry confirmed that, in adults, a high frequency of Vγ9^+^Vδ2^+^ T cells expressed PLZF, NKG2A, and CXCR3 (Figure 5F). Therefore, in adults, almost all Vγ9^+^Vδ2^+^ T cells adopt an ‘‘innate-like effector’’ program distinct from adaptive subsets.

To assess if Vγ9^+^Vδ2^+^ T cells were effector-like from birth, we analyzed cord blood samples, a setting where Vδ1^+^ T cells subsets are phenotypically highly naive and have an unfocused TCR repertoire. Cord blood Vγ9^+^Vδ2^+^ T cells displayed high levels of Eomes, HOBIT, CD161, and IL-7Rα. However, relative to adult Vγ9^+^Vδ2^+^ T cells, they had lower T-bet, HOBIT, granzyme A, and perforin expression, indicating an immature effector phenotype (Figures 5G and 5H). However, Vγ9^+^Vδ2^+^ T cells in young healthy children (≤3 years) displayed a transition to a T-bet^+^ HOBIT^+^ phenotype, increased levels of granzyme A and perforin, and a dominant CD45RO^+^ phenotype (Figures 5G and 5H). These results suggest Vγ9^+^Vδ2^+^ T cells are a dedicated effector subset at birth but transition to a more mature innate-effector program similar to that present in adults, in early life.

### Adaptive γδ T cell subsets undergo transcriptional reprogramming *in vivo*

**Figure 6.**
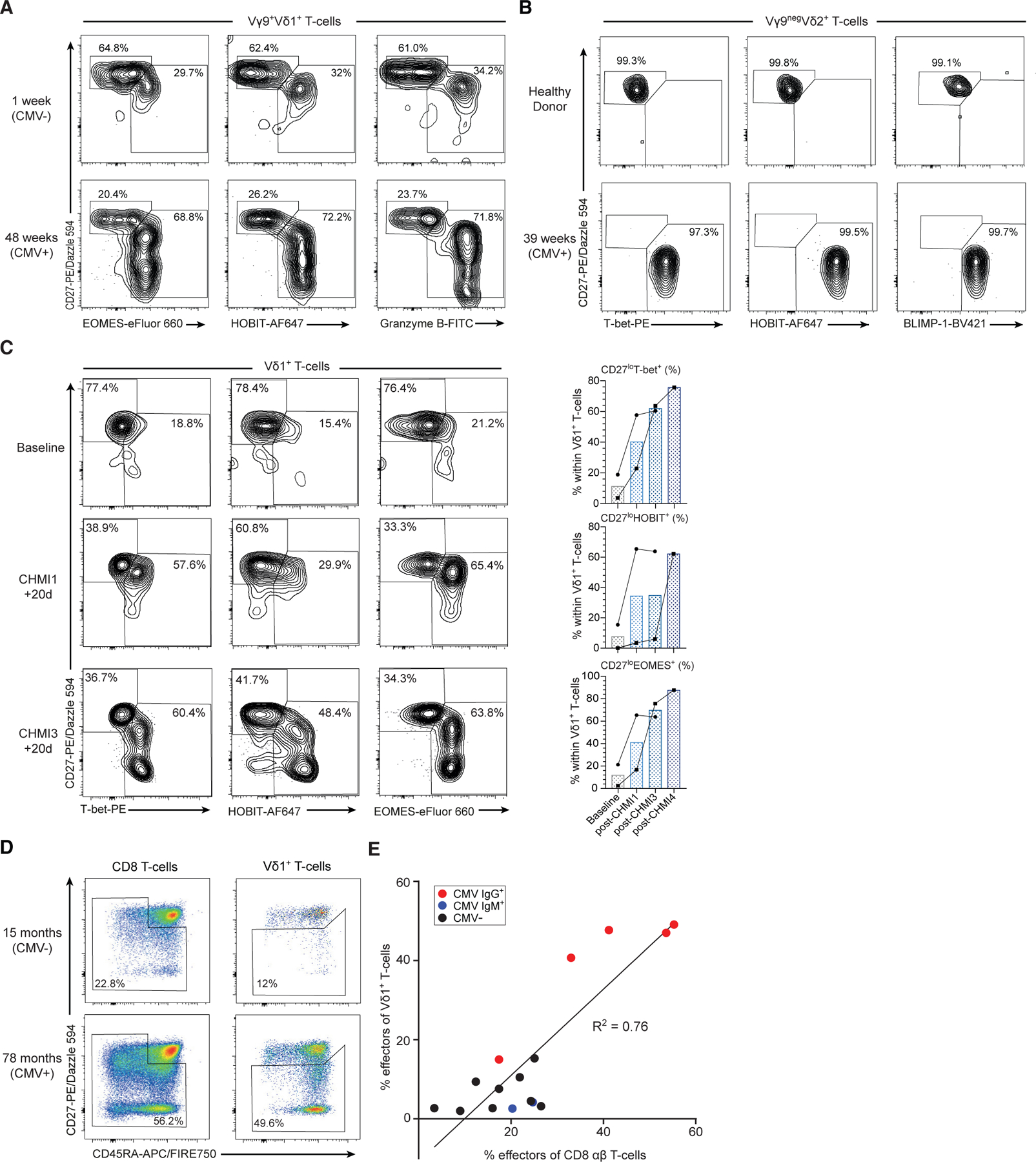
Adaptive differentiation of human γδ T cells *in vivo* (A) Transition of Vγ9^+^Vδ1^+^ T cells to a T_effector_ transcriptional program following CMV infection after kidney transplantation involving increased Eomes^+^, HOBIT^+^, and granzyme B^+^ T_effector_ cells. (B) Vγ9^neg^Vδ2^+^ T cells transition from a predominantly CD27^hi^, T-bet^neg^, HOBIT^neg^, BLIMP-1^neg^ phenotype to a CD27^lo/neg^, T-bet^+^, HOBIT^+^, BLIMP-1^+^ phenotype 39 weeks post-CMV infection. (C) Transition of Vδ1^+^ T cells from a T_naive_ to a T_effector_ transcriptional program following consecutive controlled human malaria challenge (CHMI) in two malarianaive individuals. (Left panel) Flow cytometry plots from one individual showing baseline (malaria naive), CHMI challenge 1 + 20 days after first infection and CHMI challenge 3 + 20 days and measure expression of T-bet, HOBIT, and Eomes versus CD27. (Right panel) Frequencies of T-bet^+^, HOBIT^+^, and Eomes^+^ Vδ1^+^ T cells from the same individual and second individual undergoing CHMI. (D) Assessment of Vδ1^+^ T_effector_ (CD27^lo/neg^) and CD8^+^ effector (T_CM_ + T_EM_ + T_EMRA_) cells in 16 healthy children (aged 12 months to 12 years). (E) Percentage of CD27^lo/neg^ Vδ1^+^ cells correlates with percentage of CD8^+^ effector αβ T cells (R^2^ = 0.76, Spearman coefficient = 0.73, p = 0.0017).

To assess transition from T_naive_ to T_effector_ status in adaptivelike γδ T cells *in vivo*, we examined the transcriptional phenotype of such cells before and after pathogen challenge in two separate human *in vivo* infection models; firstly CMV infection in patients undergoing kidney transplantation, and secondly repeated controlled human malaria challenge (CHMI) in malaria-naive individuals. Changes in Vδ1^+^ T cells in patients who became CMV seropositive after transplantation were consistent with an infection-induced increase in the proportion of CD27^lo/neg^ T-bet^+^ Eomes^+^ granzyme B^+^ T_effector_ cells (Figure 6A). We also examined changes in the adaptive-like Vγ9^neg^Vδ2^+^ T cell compartment. This was previously shown to undergo clonotypic expansion with concomitant phenotypic differentiation from a naive TCR-unfocused CD27^hi^ repertoire to a CD27^lo/neg^ phenotype after CMV infection (Davey et al., 2018b; Kaminski et al., 2020). Here, we demonstrate that while CMV-seronegative donor Vγ9^neg^Vδ2 T cells lacked T-bet, HOBIT and BLIMP-1, these markers were markedly upregulated upon CMV infection and seroconversion post-transplantation, alongside transition from CD27^hi^ to CD27^lo/neg^ status, suggesting transition to the T_effector_ transcriptional program (Figure 6B); in contrast, Vγ9^+^Vδ2^+^ T cells were strongly granzyme A/B^+^, T-bet^+^ and Eomes^+^ before and after CMV infection and seroconversion (Figure S6A). Secondly, CHMI with *Plasmodium falciparum* drives clonal expansion of Vδ1^+^ T cells (Rutishauser et al., 2020; von Borstel et al., 2021), providing an opportunity to assess acquisition of transcription factors in this context. At baseline, in a malaria-naive individual, 76%–78% of Vδ1^+^ T cells expressed a CD27^+^ T_naive_ phenotype (Figure 6C). Upon sequential CHMIs over 378 days, Vδ1^+^ T cells transitioned from a T_naive_ to T_effector_ subtype, as indicated by loss of CD27 and acquisition of T-bet, HOBIT, and Eomes—mirroring that seen after CMV seroconversion; HOBIT expression was most pronounced after a fourth infectious challenge (Figure 6C). Finally, in healthy children (12 months to 14 years) Vδ1^+^ T_effector_ cell levels correlated with the percentage of CD8^+^ T_effector_ cells (T_CM_ + T_EM_ + T_EMRA_) as measured by cell surface phenotype (CD27^lo/neg^ Vδ1^+^ T_effector_) or perforin expression (Figures 6D, 6E, and S6B–S6E). As expected, CMV-seropositive children had higher frequencies of Vδ1^+^ and CD8^+^ T_effector_ cells, but some CMV-seronegative children also had effector Vδ1^+^ and CD8^+^ T cells, suggesting Vδ1^+^ and CD8^+^ T cells can act as part of a coordinated adaptive immune response, which is dependent on antigen exposure. In contrast, Vγ9^+^Vδ2^+^ T cells in healthy children were perforin positive regardless of Vδ1^+^ or CD8^+^ T_effector_ cell levels or CMV status (Figures S6F and S6G).

## DISCUSSION

Evidence is emerging that an adaptive immune paradigm underpins the function of human γδ T cell subsets, including Vδ1^+^ (Davey et al., 2017, 2018a; Ravens et al., 2017; von Borstel et al., 2021) and Vγ9^neg^Vδ2^+^ T cells (Davey et al., 2018b, 2018c), both in PB and at least some solid tissues (Davey et al., 2018b; Hunter et al., 2018). Such adaptive biology likely impacts both anti-viral (Davey et al., 2018b; Dechanet et al., 1999; Ravens et al., 2017) and anti-parasite (Rutishauser et al., 2020; von Borstel et al., 2021) immunity and autoimmune responses (Hohlfeld et al., 1991; Mayassi et al., 2019; Pluschke et al., 1992). In contrast, Vγ9^+^Vδ2^+^ lymphocytes predominant in PB (Davey et al., 2018b; Willcox et al., 2018), and likely intestinal Vg4^+^ T cells (Mayassi et al., 2019; Melandri et al., 2018), appear to be more innate-like. Although some functional and clono/phenotypic changes have been observed during adaptive γδ T cell differentiation, its transcriptional and epigenetic basis (factors affecting chromatin accessibility), and how this compares with conventional αβ T cell differentiation and innate-like γδ T cell subsets, has remained unclear. Here, we used a multi-pronged approach to address this question.

Single-cell methods showed that, in adult PB, most Vδ1^+^ lymphocytes in the majority of healthy donors occupy one of two transcriptional states. These align closely with phenotypically distinct Vδ1^+^ T_naive_ and T_effector_ subtypes defined previously, which differentially express markers, such as CD27, IL7Rα, CX3CR1, perforin, and granzyme A/B (Davey et al., 2017, 2018a), and exhibit differential chromatin accessibility. Our data reveal close transcriptional parallels with conventional αβ CD8^+^ T_naive_ and CD8^+^ T_EMRA_ subsets, to which the Vδ1^+^ T_naive_ and T_effector_ subsets respectively exhibit phenotypic similarity (Davey et al., 2017, 2018a). There is a strong overlap in the transcription factors governing these naive/effector states in the Vδ1^+^ and CD8^+^ compartments. Naive Vδ1^+^ and CD8^+^ T cells are discriminated by TCF7/LEF1 expression, previously highlighted as crucial for CD8^+^ identity (Xing et al., 2016). The Vδ1^+^ T_effector_ and CD8^+^ T_EMRA_ state is distinguished by upregulation of Eomes, T-bet, and BLIMP-1, all critical to T cell effector program maintenance (Fu et al., 2017; Henning et al., 2018; Kaech and Cui, 2012; Lazarevic et al., 2013; Vieira Braga et al., 2015). Vδ1^+^ T_effector_ and CD8^+^ T_EMRA_ transcriptomes were especially closely matched, with a majority of genes increased in Vδ1^+^ T_effector_ cells versus T_naive_ cells also increased in CD8^+^ T_EMRA_ versus T_naive_ cells, reflecting upregulation of pathways such as cytotoxicity and IFN-γ production. Co-expression of HOBIT alongside T-bet/Eomes, as observed in Vδ1^+^ T_effector_ cells, is a feature of long-lived CD8^+^ T cells contributing to immunity to persistent viruses that periodically reactivate (Vieira Braga et al., 2015) and Eomes expression in CD8^+^ T cells favors long-term persistence and effector memory function (Kaech and Cui, 2012).

Conserved regulation by such critical transcription factors and involving similar downstream effector pathways strongly suggests that Vδ1^+^ T cells undergo ‘‘transcriptional reprogramming’’ upon differentiation from T_naive_ to T_effector_ status, analogous to CD8^+^ T cells. This transition likely applies to a second adaptive-like subset, Vγ9^neg^Vδ2^+^ T cells (Davey et al., 2018b), and may be a general feature of human adaptive γδ T cell differentiation. This common differentiation program suggests that PB Vδ1^+^ T_effector_ subsets may respond to intracellular pathogens, such as persistent viruses, using effector functions analogous to CD8^+^ T cells. Our results suggest that, in children, adaptive γδ subsets may often operate in parallel with adaptive CD8^+^ arms. However, such adaptive γδ T cell populations likely complement conventional αβ T cell responses by providing an alternative, MHC-unrestricted immune surveillance arm that may be particularly significant in certain recurrent or chronic pathogen infections, and conceivably tumor immunosurveillance. Consistent with this, genes involved in regulating thresholds for TCR/peptide MHC recognition signaling thresholds were among the rare genes differentially expressed between the Vδ1^+^ T_effector_ and CD8^+^ T_EMRA_ subsets, with CD5, THEMIS, and SET1 all enriched in CD8 T_EMRA_ cells.

Despite similarities between Vδ1^+^ T cell and CD8^+^ T cell differentiation, we note some differences. Firstly, Vδ1^+^ T_naive_ and CD8^+^ T_naive_ subsets clearly exhibit greater differences than is evident between Vδ1^+^ and CD8^+^ T_effector_/T_EMRA_ subsets, which are exceedingly concordant. These include an enhanced level of some NKR transcripts in Vδ1^+^ T_naive_ cells relative to CD8^+^ T_naive_ cells. Understanding the transcriptional, epigenetic, and functional significance of this is a key future aim, and may clarify signals that trigger Vδ1^+^ T_naive_ to T_effector_ cell differentiation. Secondly, Vδ1^+^ lymphocytes clustered into only two subpopulations, whereas CD8^+^ T cells adopt several phenotypically distinct memory states. This may denote a fundamental difference in differentiation, or may alternatively reflect our study’s limitations.

A striking feature of several transcription factors differentially expressed between Vδ1^+^ T_naive_ and T_effector_ subsets is their strong regulation by TCR triggering in CD8^+^ T cells. TCF7/LEF1 is lost from CD8^+^ T_naive_ cells upon TCR-dependent antigenic stimulation and priming (Willinger et al., 2006), and T-bet and Eomes are upregulated upon TCR signaling in CD8^+^ T cells, with amplification via cytokine receptor signaling (Kaech and Cui, 2012). Several observations suggest that adaptive γδ T cell differentiation is similarly driven or initiated by TCR triggering. Firstly, BLIMP-1, T-bet, and Eomes upregulation in T_naive_ Vδ1^+^ cells was dependent on CD3 stimulation, consistent with γδ TCR ligation triggering upregulation of transcription factors linked to the T_effector_ state. These findings were mirrored in CD8^+^ αβ T cells, and HOBIT was upregulated on neither Vδ1^+^ nor CD8^+^ T_naive_ populations, consistent with its discordant regulation relative to BLIMP-1 after antigen stimulation (Kragten et al., 2018). Given the long duration of this assay, such CD3-driven upregulation of T_effector_ transcription factors may result from either direct signaling downstream of the TCR, or indirect effects, such as TCR-triggered cytokine production, or a combination of both. Secondly, the T_naive_ to T_effector_ transition is tightly linked to clonotypic expansion: consistent with single-cell data presented here, both Vδ1^+^ and Vγ9^neg^Vδ2^+^ T_naive_ cells are clonotypically diverse and unfocused, whereas clonally expanded TCRs are only detected in T_effector_ subsets (Davey et al., 2017, 2018b). Finally, in CMV and malaria infection models, pathogen exposure elicited Vδ1^+^ (and for CMV also Vγ9^neg^Vδ2^+^) clonal expansions (Davey et al., 2018b, 2018c; Ravens et al., 2017; Rutishauser et al., 2020; von Borstel et al., 2021), and drove phenotypic differentiation (Davey et al., 2018b, 2018c; Rutishauser et al., 2020; von Borstel et al., 2021). Collectively, these observations suggest that, as for conventional αβ T cells, cognate γδ TCR antigen recognition plays a critical role in triggering adaptive γδ T cell differentiation. The fact that T_effector_ populations display upregulated and expedited cytotoxicity/cytokine secretion relative to T_naive_ cells, retain CD3/TCR sensitivity, and exhibit long-term persistence *in vivo*, suggests ongoing potential for γδ TCR ligand recognition during secondary/tertiary responses.

Previous studies on transcriptional regulation of T cell differentiation have focused largely on conventional αβ T cells. This study outlines comprehensively the transcriptional basis for adaptive γδ T cell biology. The results are in accordance with, but extend substantially, our previous work on Vδ1^+^ T_naive_ and T_effector_ cells (Davey et al., 2017, 2018a). These studies established phenotypic and clonotypic changes consistent with some kind of adaptive differentiation, but did not define underlying transcriptional paradigms and their alignment with conventional αβ T cell differentiation. Many previous studies have assessed Vδ1^+^ T cells *en masse*, an example being Gutierrez-Arcelus et al. (2019), which positioned Vδ1^+^ T cells on an ‘‘innateness gradient.’’ In light of our results, caution should be exerted in such interpretations, as factors such as age and infectious status heavily influence the balance between T_naive_ and T_effector_ Vδ1^+^ populations and are major confounding factors, limiting the utility and validity of such a gradient; the innateness scale proposed is also likely to overlap largely with effector program signatures. Also, expression of markers, such as NKG2D, on Vδ1^+^ T cells is sometimes interpreted as denoting an innate-like biology (Wu et al., 2019), whereas NKG2D is upregulated on both Vδ1^+^ T_effector_ and CD8^+^ T_EMRA_ cells, and may alternatively reflect the presence of adaptive Vδ1^+^ T_effector_ populations.

Our results confirm that, relative to adaptive subsets, Vγ9^+^Vδ2^+^ T cells adopt a fundamentally different and innate-like immunobiology. Unlike Vδ1^+^ T cells, the adult Vγ9^+^Vδ2^+^ T cell subset lacked a distinct T_naive_ subpopulation devoid of effector markers, and universally expressed T-bet and Eomes. The transcriptional profile of Vγ9^+^Vδ2^+^ T cells unmistakably denoted a type of T_effector_ status based on expression of granzyme A/B, perforin, and T-bet/Eomes. However, elements of this profile were notably different from both Vδ1^+^ and CD8^+^ T_effector_ cells, including expression of PLZF, a transcription factor linked with innate-like T cells (Fergusson et al., 2014; Kovalovsky et al., 2008; Kreslavsky et al., 2009; Savage et al., 2008), NKG2A and GZMK, both expressed by NK cells (Bratke et al., 2005; Brooks et al., 1997), and distinct cytokine and chemokine receptor expression patterns. A caveat is that we assessed Vγ9^+^Vδ2^+^ T cells *en masse*, whereas some other studies (Ryan et al., 2016; Wragg et al., 2020) have defined phenotypically distinct subsets present in multiple individuals. The central memory phenotype of Vγ9^+^Vδ2^+^ T cells present in most donors is consistent with profiles observed by Ryan et al. (2016); we observe NKG2A expression on most Vγ9^+^Vδ2^+^ cells in all donors, consistent with Wragg et al.’s findings (2020). Also, we observe markers linked with innate-like T cells (e.g., NKG2A, PLZF, C/EBPd), consistent with previous work (Gutierrez-Arcelus et al., 2019), which indicated similarities between Vγ9^+^Vδ2^+^, MAIT, and iNKT transcriptional profiles. While we did not directly compare NK transcriptional profiles, Pizzolato et al. (2019) detected a subpopulation of γδ T cells clustering with NK cells and this may align with the Vγ9^+^Vδ2^+^ subset. In contrast to Vδ1^+^ T cells, our results suggest a predisposition to effector status from birth and maturation early in life, since Vγ9^+^Vδ2^+^ T cell expression of Eomes and HOBIT was evident in cord blood, and universal by <3 years, consistent with recent findings (Papadopoulou et al., 2020). Such an innate-like paradigm is likely facilitated by the semi-invariant Vγ9^+^Vδ2^+^ TCR repertoire present from birth, featuring prevalence of public Vγ9^+^ clonotypes (Davey et al., 2018b; Willcox et al., 2018). While our results support post-natal maturation of Vγ9^+^Vδ2^+^ T cells (Ryan et al., 2016; Wragg et al., 2020), they also suggest that, from early life onward, changes reflect modification of an innate-effector transcriptional profile/phenotype, rather than ongoing potential for the more radical transcriptional reprogramming that applies to adaptive γδ T cell subsets.

In summary, we show that certain γδ T cell subsets can, throughout life and in response to specific immunological stimuli, undergo adaptive ‘‘transcriptional reprogramming’’ akin to changes in CD8^+^ T cells upon antigen-induced differentiation. This paradigm applies to Vδ1^+^ T cells present in PB and diverse tissues (Davey et al., 2017, 2018a; Hunter et al., 2018; Ravens et al., 2017) and the less prevalent Vγ9^neg^Vδ2^+^ subset (Davey et al., 2018b, 2018c). We suggest that adaptive differentiation drives inflammatory/cytotoxic γδ T cell responses to specific immune challenges in diverse biological scenarios. These likely include beneficial responses to pathogen infection (Davey et al., 2018a, 2018c), including CMV (Davey et al., 2018b; Ravens et al., 2017), EBV (Farnault et al., 2013; Fujishima et al., 2007), and malaria (Rutishauser et al., 2020; von Borstel et al., 2021). They likely complement conventional adaptive αβ T cell immunity, by permitting pathogen-specific responses to microenvironmental niches with compromised MHC expression, either resulting from viral immune evasion (for CMV and EBV), infection of cells devoid of MHC (e.g., red blood cells in the case of *P. falciparum*), or conceivably class I MHC loss during tumor development and immune evasion. However, adaptive γδ responses may sometimes promote immunopathology, as in autoimmune myositis driven by clonotypically focused Vγ9^neg^Vδ2^+^ T cells apparently T_effector_ in phenotype (Hohlfeld et al., 1991; Pluschke et al., 1992). A recent study reported that, in coeliac disease, the intestinal IEL population is enriched with inflammatory, T-bet^+^, clonotypically focused Vδ1^+^ cells that may be antigen driven, aligning with the adaptive γδ T cell differentiation paradigm outlined here (Mayassi et al., 2019). Future studies will no doubt extend the relevance of this adaptive paradigm to different settings. Understanding the transcriptional regulation of γδ T cell differentiation may also facilitate development of γδ T cell immunotherapy approaches in infectious disease and cancer.

### Limitations of the study

We highlight three study limitations. Firstly, we focus on a modest number of individuals and on a limited number of γδ T cells in PB; a wider set of Vδ1^+^ T cell differentiation states therefore likely exist. Future studies will no doubt elaborate on our findings by exploring larger cohorts and/or cell numbers, and assessing γδ T cells in diverse scenarios, including active infection, and different tissues. Consistent with this, we recently defined a tissue-resident-like hepatic Vδ1^+^ subset phenotypically and functionally distinct from PB subsets (Hunter et al., 2018). Secondly, we used CD8^+^ T_EMRA_ cells as a conventional effector T cell comparator; however, more marked differences between Vδ1^+^ T_effector_ cells and other CD8^+^ T cell subtypes may exist; we did not directly compare transcriptional profiles of CD4^+^ T cell populations, and alignment of Vδ1^+^ to CD4^+^ T cell differentiation warrants further study. Finally, despite strong similarities between Vδ1^+^ and CD8^+^ T cell subsets, our results do not exclude the possibility that, aside from MHC-unrestricted target cell recognition, adaptive γδ T cell populations may exhibit substantial functional differences to conventional counterparts, such as in their dynamics or relative importance of TCR-independent regulatory axes (e.g., mediated via NKRs).

## STAR★METHODS

### RESOURCE AVAILABILITY

#### Lead contact

Further information and requests for resources and reagents should be directed to and will be fulfilled by the lead contact, Benjamin Willcox (b.willcox@bham.ac.uk).

#### Materials availability

This study did not generate new unique reagents.

#### Data and code availability

Raw data utilised in this study has been uploaded to the SRA database, under the accession code PRJNA562324 and the project title ‘‘Epigentic and transcriptional profiling of human gamma delta T cells’’.

This paper does not report any original code.

Any additional information required to reanalyze the data reported in this paper is available from the lead contact upon request.

### EXPERIMENTAL MODEL AND SUBJECT DETAILS

#### Ethical approval and samples

Peripheral blood samples were obtained from healthy donors or from buffy packs. All donors provided written informed consent for sample collection and subsequent analysis; project approval for this aspect of the study was granted by the NRES Committee West Midlands ethical board (REC reference 14/WM/1254) or for buffy packs by the Australian Red Cross (ARC) Lifeblood ethics committee and Monash University Human Research Ethics Committee (Reference, 19,488 and 14,487). Samples from patients undergoing renal transplantation were obtained at the Academic Medical Centre, Amsterdam; the medical ethics committee of the Academic Medical Center, Amsterdam, approved this arm of the study and all subjects provided written informed consent in accordance with the Declaration of Helsinki. Umbilical cord blood units were obtained from the Anthony Nolan Cell Therapy Centre Nottingham (ANCTC) under generic tissue bank ethics held by ANCTC and extended to the researchers under a material transfer agreement (MTA). Paediatric healthy volunteer samples were obtained as part of the TRICICL study (Tracking the Immune changes in children with cancer) with IRAS number 233593 and REC reference 17/WM/0453; health research authority and ethics approval was obtained from South Birmingham research ethics committee. Peripheral blood samples from two subjects participating in the controlled human malaria infection (CHMI) study were also included. Heparinized venous blood was collected at baseline (before infection), and twenty days (d) after the first (CHMI1), third (CHMI3), and for one subject, fourth *P. falciparum* infection (CHMI4). Subjects were infected by bites of mosquitos carrying *P. falciparum* (strain NF54) and were treated with anti-malarial drugs (Malarone and CoArtem as a secondary treatment) based upon the detection of two unquestionable parasites by blood smear. Both subjects gave written informed consent and the study was approved by the medical ethics committee of the University of Maryland, Baltimore (ClinicalTrials.gov, NCT03014258).

### METHOD DETAILS

#### T-cell isolation, culture, activation

PBMC were isolated from heparinised venous blood or buffy packs by using lymphoprep© (Stem Cell Technologies) density gradients. Resulting PBMC were frozen in liquid nitrogen and thawed to be used for subsequent experiments. Cells were sorted on an ARIA III Fusion Cell Sorter (BD Bioscience) at the University of Birmingham or FlowCore, Monash University. PBMC and purified T-cells were cultured in RPMI-1640 medium (Invitrogen) supplemented with 2 mM L-glutamine, 1% sodium pyruvate, 50 μg/mL penicillin/streptomycin (Invitrogen) and 10% fetal calf serum (Sigma). In total, Vδ1^+^ T_effector_ (CD27^lo/neg^) populations were sorted from 6 healthy donors, and Vδ1^+^ T_naïve_ (CD27^hi^) from 3, CD8^+^ T_naïve_ from 7, and CD8^+^ T_EMRA_ populations were obtained from 9, with comparator Vδ2^+^ populations obtained from 2 individuals (Davey et al., 2017).

#### Antibodies and flow cytometry

For phenotypic analysis and cell sorting, freshly isolated, frozen PBMC or cultured cells were labelled with Zombie Aqua viability dye (Biolegend), and Vδ1^+^ and Vγ9^+^Vδ2^+^ populations were identified with anti-CD3 (UCHT1; Biolegend), TCR ab (IP26; ThermoFisher), TCR Vδ1 (REA173; Miltenyi), TCR Vδ2 (123R3; Miltenyi), TCR Vγ9 (IMMU360; Beckman Coulter), CD27 (M-T271; Biolegend) and CD45RA (HI100; Biolegend) and various combinations of CXCR3 (GO25H7), CX_3_CR1 (2A9–1), CD127 (A019D5), CD45RO (UCHL1), CD161 (HP-3G10), all Biolegend; or NKG2A (Z199; Beckman Coulter). Populations were gated as outlined previously (Davey et al., 2017). For intracellular staining, cells were fixed in IC Fixation buffer (eBioscience) after surface antibody staining and finally stained in Permeabilisation Buffer (eBioscience) with antibodies directed against Granzyme A (CB9), Granzyme B (GB11), Perforin (B-D48), TCF7 (7F11A19), all Biolegend; EOMES (WD1928, Invitrogen); Blimp-1 (6D3) and Hobit (Sanquin-Hobit/1), both BD Biosciences; and T-bet (eBio4B10) and PLZF (21F7), both eBioscience. Cells were acquired on a Fortessa (BD Biosciences) and data analysed using FlowJo V10.1 (TreeStar & BD Biosciences). For detection of intracellular cytokines, cells were stimulated with 10 ng/mL PMA and 1 μg/mL ionomycin (both Sigma) for 1 h before adding 5 μg/mL brefeldin A (ThermoFisher) and 2 μM monensin (BD Biosciences) to the cultures for 4 h prior to harvesting. Surface-stained cells were labeled using the Fix & Perm kit (ThermoFisher) and monoclonal antibodies against IFNγ (4S.B3), TNFα (mAb11), CCL5 (VL1), IL-2 (MQ1–17H12), IL-17A (BL168), GM-CSF (BVD2–21C11), all Biolegend.

#### Single cell transcriptomics analysis

Single CD3^+^ TCR-αβ ^neg^ Vδ1^+^ T-cells (CD27^hi^ or CD27^lo/neg^) from three donors were sorted directly into the wells of 96 well PCR plates containing 2 μL of 0.2% Triton X-100 (Sigma) with 2 U/μL recombinant RNase inhibitor (Takara Bio) and frozen at −80°C. cDNA was generated using the SmartScribe Reverse Transcriptase (Takara Bio Cat. 639,538), preamplified using the KAPA HiFi Hot Start Ready Mix, and sequencing libraries generated with the Nextera XT kit, following the protocol of Picelli et al. (2014).

Gene expression was quantified using Salmon (Patro et al., 2017), exporting transcripts per million (TPM) (version 0.6.0). Downstream analysis was completed using the *Seurat* package (v 2.3.4) (Butler et al., 2018) in R (v 3.5.3) (Team, 2000). Ensembl genes were mapped to HUGO gene symbols using the *biomaRt* package for R(Durinck et al., 2009). Upon conversion, 1,483 genes were not annotated and were discarded. Where symbols were not available, ensembl IDs were used. Genes were subsequently re-scaled to sum to 1 million and summated for those Ensembl genes which resolved to the same gene name. Three cells were deemed outliers by PCA analysis, and genes were filtered according to the Seurat package user guide. Data were separated into two clusters using the FindClusters() function under default conditions other than a resolution of 0.5. Data were displayed using PCA dimension reduction. To determine the number of significant principal components, both an Elbow plot and a JackStraw plot (100 replications) were constructed. An ‘elbow’ was observed at PC2, and PC1 and PC2 were deemed significant by JackStraw analysis. PC1 was the most significant principal component and was, therefore, further investigated between the clusters. Differential gene expression utilised a bimodal test within *Seurat*, an FDR cut-off of 0.05 (Bonferroni corrected), and a Log(Fold Change) threshold of 0.25. Interclone differences were calculated by a one-versus-many approach. The largest expanded clones for each donor (A, E, and K) were taken and individually compared to the others i.e. A versus E and K.

#### Reconstruction of γδ TCR sequences from single cell RNAseq

γδ TCR sequences were reconstructed from single cell transcriptomes, using TraCeR as previously described (Stubbington et al., 2016). TCRγ and TCRδ reference files were constructed from IMGT (Lefranc et al., 2009) reference sequences using the ‘build’ command of TraCeR. Given previous observations of long junctional regions for TCRδ (Davey et al., 2017; Hunter et al., 2018), the ‘max_junc_len’ parameter was increased to 100.

#### Bulk RNAseq analysis

Naive (CD27^hi^) and effector (CD27^lo/neg^) Vδ1^+^ T-cells or CD8^+^ naive (CD27^hi^ CD45RA^hi^) and T_EMRA_ (CD27^–^CD45RA^hi^) cells were sorted into RNAlater (Sigma Aldrich). Total RNA was isolated using the RNAmicro kit (Qiagen) according to the manufacturer’s instructions and RNA sequencing was performed by Genomics Birmingham (Birmingham, UK) or the Medical Genomics facility (MHTP, Clayton, Australia). FASTQ files were aligned to hg38 using HISAT2 (version 2.1.0) (Kim et al., 2015) under default conditions for paired-end alignment (average alignment of 90.90%). Reads were indexed using SAMtools (version 1.4) (Li et al., 2009) and counts were generated from the bam files with the *Rsubread* package (version 1.32.2) (Liao et al., 2013) in R (version 3.5.1) using the inbuilt annotation for the hg38 genome assembly. Batch effects were removed from the count data using the *CountClust* package for R (Dey et al., 2017; Taddy, 2012). Outliers were determined using PCA and the *arrayQualityMetrics* R package (Kauffmann et al., 2009) in accordance with outlier removal peformed by Guinney et al. (Guinney et al., 2015). Downstream analyses utilised the *edgeR* package (version 3.24.2) (Robinson et al., 2010) (McCarthy et al., 2012). As suggested in the *edgeR* user guide, reads were filtered based on counts per million (CPM). Using a threshold of 6–7 reads in the sample with the smallest library, the CPM scaling factor was calculated. Genes with a CPM above 3.04 in at least two samples (smallest cell population) were kept for further analysis as suggested. This filtering step removed 15,768 lowly expressed transcripts, including 5,640 that were 0 in all 27 samples. Filtered reads were passed to the plotMDS() function, using all available reads for multidimensional scaling. Utilisation of *voom* transformation allowed the use of the *limma* (version 3.38.3) package for differential gene expression analysis (Law et al., 2014; Ritchie et al., 2015). Genes were adjusted using the Benjamin-Hochberg method, as is the default and were considered significant if p < 0.05. Geneset enrichment analysis utilised the GO and KEGG database of terms, using the camera method (*limma* package). Genesets with less than 10 or more than 300 genes were discarded. The output of geneset enrichment analysis were visualised using Cytoscape (Shannon et al., 2003) (version 3.7.1) and the *RCy3* package (version 2.2.6) (Ono et al., 2015). A similarity threshold of 0.25 was based on the Jaccard coefficient, and only genesets passing the 0.001 q value threshold were plotted. The graphic output used a force-directed layout. Heatmaps were constructed utilising the *gplots* R package (Warnes et al., 2020), using Euclidean distance and Ward linkage.

#### ATACseq analysis

Vδ1^+^ T-cells, CD8^+^ T-cells and Vγ9^+^Vδ2^+^ T-cells (20,000 of each population) were sorted into MACS buffer and cells were pelleted by centrifugation at 400 3 g. ATACseq libraries were generated using the Nextera DNA Library Prep Kit (Illumina Cat. FC-121–1030) in the presence of 0.01% digitonin (Promega Cat. G9441) and amplified using NEBNext High-Fidelity 2× PCR Master Mix (New England Labs Cat #M0541) for 9–13 cycles (Buenrostro et al., 2015). Amplified libraries were purified using Ampure beads (Beckman Coulter) and resuspended in 0.1× TE before quantification on Tapestation (Agilent), pooling and sequencing using the NextSeq 500/550 High Output kit v2.5 (Illumina) at Genomics Birmingham (Birmingham, UK). Paired end FASTQ files were aligned to the GRCh38 genome assembly using Bowtie2 (Langmead and Salzberg, 2012) (version 2.3.5.1), and sorted and indexed using SAMtools (version 1.9). Average number of fragments was 34,056,451, with 89.09% alignment to the reference genome. Samples had a relatively low alignment to the mitochondrial genome (4.21%) Duplicate sequences due to PCR were removed using Picard (Broad Institute, 2019) (version 2.21.1) and peaks were called using MACS2 (Zhang et al., 2008) (version 2.2.7.1) using settings of –shift 100 and –extsize 200. An average of 55,236 peaks were detected. HOMER (Heinz et al., 2010) (version 4.11) was used to find consensus peaks amongst the samples, using a distance argument of 100. 21,208 consensus peaks were found of which counts were subsequently normalised to 1e7 per sample and annotated using HOMER with a –size 400. Raw bam files were opened in IGV Genomics Viewer (version 2.8.12), and group autoscaled for visualisation.

#### CD3/CD28/IL-2 stimulation assays

For assessing TCR-mediated regulation of transcription factor expression, enriched Vδ1^+^ and CD8^+^ T-cells were used. Individuals with a substantial proportion of T_naive_ Vδ1^+^ T-cells were prioritised for these experiments. Vδ1^+^ T-cells were negatively enriched from PBMC through depletion of αβ T-cells, B-cells, Vδ2^+^ T-cells, and monocytes by using a cocktail of APC-conjugated anti-αβ TCR (clone IP26, Thermo Scientific), CD19 antibody (clone HIB19, TONBO biosciences), Vδ2 TCR antibody (clone 123R3, Miltenyi Biotec), and CD14 antibody (clone 61D3, TONBO bioscience). CD8^+^ T-cells were negatively enriched from PBMC through the depletion of CD4^+^ T-cells, B-cells, Vδ2^+^ T-cells, Vδ1^+^ T-cells and monocytes by using a cocktail of APC-conjugated anti-CD4 (RPA-T4, Cy7-conjugated, BD Pharminogen), anti-CD19 antibody (clone HIB19, TONBO biosciences), anti-Vδ2 TCR antibody (clone 123R3, Miltenyi Biotec), anti-Vδ1 TCR antibody (clone REA173, Miltenyi Biotec), and anti-CD14 antibody (clone 61D3, TONBO bioscience). The APC-labelled cells in both settings were eliminated using EasySep Human APC Selection Kit (Stemcell Technologies). Enriched T-cells were then incubated at 37°C in the presence of plate-bound anti-CD3 antibodies (OKT-3, Biolegend) or 100 U/mL IL-2 (Peprotech) for 6 days. The expression level of relevant transcription factors in the Vδ1^+^ T_naïve_ (CD27^hi^) and CD8^+^ T_naïve_ subsets were measured on day 0 (unstimulated) and on day 6 of culture using flow cytometry. Data were analysed using FlowJo (version 10) software.

### QUANTIFICATION AND STATISTICAL ANALYSIS

Tabulated data were analysed in Graphpad PRISM 7 (Graphpad Software Inc). Each dataset was assessed for normality using Shapiro-Wilko normality tests. Differences between groups were analyzed using one-way ANOVA with Holm-sidak’s or Tukey’s post-tests for normally distributed data; two-way ANOVA was used when comparing groups with independent variables. *p < 0.05, **p < 0.01, ***p < 0.001 and ****p < 0.0001. Correlation was assessed with Spearman correlation, and R^2^ values are reported.

## Supplementary Material

1

2

3

4

5

6

## Figures and Tables

**Table:** KEY RESOURCES TABLE

REAGENT or RESOURCE	SOURCE	IDENTIFIER
Antibodies		

Anti-human CD3, clone UCHT1	Biolegend	Cat# 300460
TCR αβ, clone IP26	ThermoFisher	Cat#535531
TCR Vδ1, clone REA173	Miltenyi	Cat#130–118-362
TCR Vδ2, clone 123R3	Miltenyi	Cat#130–095-796
TCR Vγ9, clone IMMU360	Beckman Coulter	Cat#A63663
CD27, clone M-T271	Biolegend	Cat#356422
CD45RA, clone HI100	Biolegend	Cat# 304151
CXCR3, clone GO25H7	Biolegend	Cat#353705
CX_3_CR1, clone 2A9–1	Biolegend	Cat#341626
CD127, clone A019D5	Biolegend	Cat#351334
CD45RO, clone UCHL1	Biolegend	Cat# 304237
CD161, clone HP-3G10	Biolegend	Cat# 339903
NKG2A, clone Z199	Beckman Coulter	Cat#A60797
Granzyme A, clone CB9	Biolegend	Cat#507204
Granzyme B, clone GB11	Biolegend	Cat#515403
Perforin, clone B-D48	Biolegend	Cat#353305
TCF-7, clone 7F11A19	Biolegend	Cat#655203
EOMES, clone WD1928	ThermoFisher	Cat #50–4877-42
Blimp-1, clone 6D3	BD Biosciences	Cat#565276
Hobit, clone Sanquin-Hobit/1	BD Biosciences	Cat#566250
T-bet, clone eBio4B10	ThermoFisher	Cat#12–5825-82
PLZF, clone 21F7	ThermoFisher	Cat#12–9320-82
IFNγ, clone 4S.B3	Biolegend	Cat#502511
TNFα, clone mAb11	Biolegend	Cat#502935
CCL5, clone VL1	Biolegend	Cat#515503
IL-2, clone MQ1–17H12	Biolegend	Cat#500347
IL-17A, clone BL168	Biolegend	Cat#512315
GM-CSF, clone B VD2–21C11	Biolegend	Cat#502305
CD19, clone HIB19	TONBO biosciences	Cat#20–0199-T100
CD14, clone 61D3	TONBO biosciences	Cat#20–0149-T100
CD3, clone OKT-3	Biolegend	Cat#317326

Biological samples		

Healthy donor peripheral blood mononuclear cells		This study

Chemicals, peptides, and recombinant proteins		

Triton X-100	Sigma	Cat#X100
recombinant RNase inhibitor	Takara Bio	Cat#2313A
RNAlater	Sigma	Cat #R0901
digitonin	Promega	Cat# G9441
Recombinant IL-2	Peprotech	Cat# 200–02
RPMI-1640	ThermoFisher	Cat#21875034
L-Glutamine	ThermoFisher	Cat#25030081
Sodium pyruvate	ThermoFisher	Cat#11360070
penicillin/streptomycin	ThermoFisher	Cat#15070063
Fetal Calf serum	Sigma	Cat#F7524
Permeabilisation Buffer	ThermoFisher	Cat#00–8333-56
PMA	Sigma	Cat#P1585
Ionomycin	Sigma	Cat#I0634
Brefeldin A	ThermoFisher	Cat#00–4506-51
Monensin	BD Biosciences	Cat#554724

Critical commercial assays		

SmartScribe Reverse Transcriptase	Takara Bio	Cat#639538
KAPA HiFi Hot Start Ready Mix	Fisher Scientific	Cat#50–196-5217
Nextera XT kit	Illumina	Cat#CFC-131–1024
RNeasy Plus Micro kit	Qiagen	Cat#74034
Nextera DNA Library Prep Kit	Illumina	Cat#FC-121–1030
NextSeq 500/550 High Output kit v2.5	Illumina	Cat#20024907
EasySep Human APC Selection Kit	Stemcell Technologies	Cat#17661

Deposited data		

Epigenetic and transcriptional profiling of human gamma delta T cells	This study	SRA database accession code PRJNA562324
Human genome build (GRCh38/hg38)	Genome Reference Consortium	https://www.ncbi.nlm.nih.gov/grc/human

Software and algorithms		

FlowJo version 10	FlowJo LLC	https://www.flowjo.com/
GraphPad Prism version 8.0.2	GraphPad Software LLC	https://www.graphpad.com
R version 3.5.1/3.5.3	R Project	https://www.r-project.org/
Salmon version 0.6.0	Patro et al. (2017)	https://salmon.readthedocs.io/en/latest/salmon.html
TraCeR	Stubbington et al. (2016)	https://doi.org/10.1038/nmeth.3800
HISAT2 version 2.1.0	Daehwankim lab	https://daehwankimlab.github.io/hisat2/
SAMTools version 1.4 & version 1.9	Li et al. (2009)	http://www.htslib.org/
Cytoscape version 3.7.1	Shannon et al., (2003)	https://cytoscape.org/
Bowtie2 version 2.3.5.1	Langmead and Salzberg (2012)	http://bowtie-bio.sourceforge.net/bowtie2/index.shtml
Picard version 2.21.1	GATK	https://sourceforge.net/projects/picard/
MACS2 version 2.2.7.1	Zhang et al. (2008)	https://pypi.org/project/MACS2/
HOMER version 4.11	University of California San Diego	http://homer.ucsd.edu/homer/
IGV Genomics Viewer version 2.8.12	Broad Institute (2019)	https://software.broadinstitute.org/software/igv/
Seurat version 2.3.4 (R package)	Butler et al. (2018)	https://satijalab.org/seurat/
edgeR version 3.24.2 (R package)	Robinson et al. (2010) McCarthy et al. (2012)	https://bioconductor.org/packages/release/bioc/html/edgeR.html

## Highlights

- Vδ1^+^ T cells undergo transcriptional reprogramming from T_naive_ to T_effector_ status
- Vδ1^+^T_effector_ cells are highly transcriptionally similar to CD8^+^ T_EMRA_ cells
- CD3 ligation of Vδ1^+^T_naive_ cells induces T_effector_-linked transcription factors
- Infections drive transition from a Vδ1^+^T_naive_ to T_effector_ transcriptional profile
