# Supplementary material for: Transcriptional profiling of human Vδ1 T cells reveals a pathogen-driven adaptive differentiation program
Source: Cell Rep. Author manuscript; Available in PMC 2022 Oct 5. (PMC9533230; doi:10.1016/j.celrep.2022.110858)
Supplement: 1 [file NIHMS1810678-supplement-1.pdf]

Cell Reports, Volume *39*

Supplemental information

Transcriptional profiling of human Vδ1 T cells reveals a pathogen-driven adaptive differentiation program

Jack L. McMurray, Anouk von Borstel, Taher E. Taher, Eleni Syrimi, Graham S. Taylor, Maria Sharif, Jamie Rossjohn, Ester B.M. Remmerswaal, Frederike J. Bemelman, Felipe A. Vieira Braga, Xi Chen, Sarah A. Teichmann, Fiyaz Mohammed, Andrea A. Berry, Kirsten E. Lyke, Kim C. Williamson, Michael J.T. Stubbington, Martin S. Davey, Carrie R. Willcox, and Benjamin E. Willcox

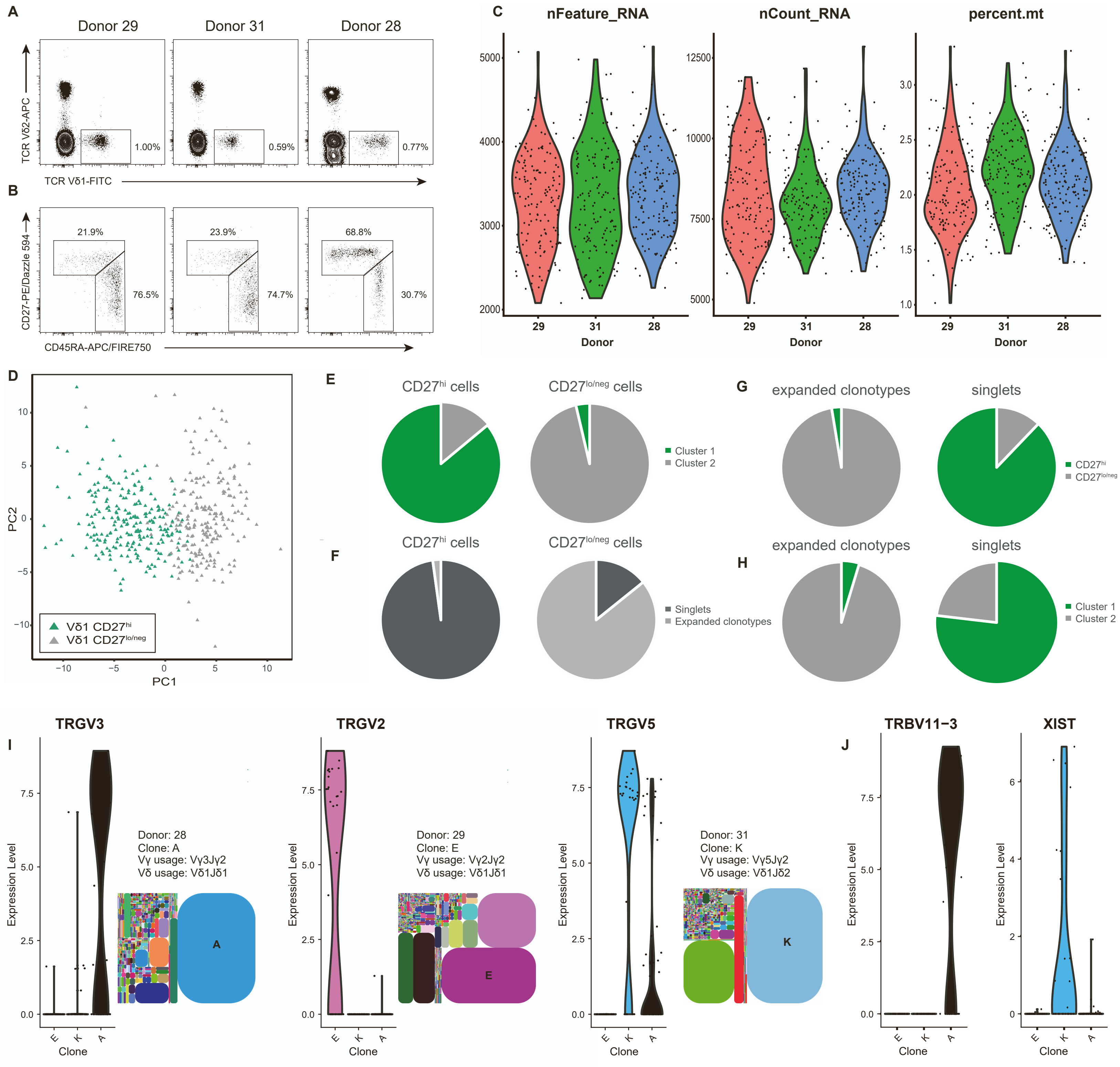
A
Donor 29
Donor 31
Donor 28
TCR Vδ2-APC
TCR Vδ1-FITC
1.00%
0.59%
0.77%
B
CD27-PE/Dazzle 594
CD45RA-APC/FIRE750
21.9%
76.5%
23.9%
74.7%
68.8%
30.7%
C
nFeature_RNA
nCount_RNA
percent.mt
Donor
D
PC1
PC2
Vδ1 CD27hi
Vδ1 CD27lo/neg
E
F
CD27hi cells
CD27lo/neg cells
Cluster 1
Cluster 2
Singlets
Expanded clonotypes
G
H
expanded clonotypes
singlets
CD27hi
CD27lo/neg
I
TRGV3
TRGV2
TRGV5
Expression Level
Clone
Donor: 28
Clone: A
Vγ usage: Vγ3Jγ2
Vδ usage: Vδ1Jδ1
Donor: 29
Clone: E
Vγ usage: Vγ2Jγ2
Vδ usage: Vδ1Jδ1
Donor: 31
Clone: K
Vγ usage: Vγ5Jγ2
Vδ usage: Vδ1Jδ2
J
TRBV11−3
XIST

Figure S1

Figure S1. Single cell transcriptomic analysis of peripheral blood Vδ1$^+$ T-cells. Related to Figure 1.

(A) Percentage of Vδ1$^+$ T-cells of CD3$^+$ T-cells, and **(B)** Phenotype of Vδ1$^+$ T-cells sorted for single cell RNAseq from three donors. Cells were sorted as CD27^{hi} or CD27$^{lo/neg}$ as indicated. **(C)** Number of genes found (nFeature), total number of transcripts (nCount_RNA), and percentage mitochondrial reads (percent.mt) in Vδ1$^+$ T-cell samples from each donor. **(D)** Principal component analysis was performed on single cell RNA sequencing data from FACS-sorted Vδ1 cells taken from three donors. Identity from FACS-results were used to colour the cells. This displayed a 91.1% concordance with identities determined through clustering. **(E)** Proportion of cells sorted as CD27^{hi} assigned to cluster 1 (197 cells; 86.0%) or cluster 2 (32 cells; 14.0%); or sorted as CD27$^{lo/neg}$ that were assigned to cluster 1 (8 cells; 3.7%) or cluster 2 (210 cells; 96.3%). **(F)** Proportion of cells sorted as CD27^{hi} expressing singlet TCRs (found only once; 224 cells; 97.8%) or expanded clonotypes (found more than once; 5 cells; 2.2%) or sorted as CD27$^{lo/neg}$ expressing singlet TCRs (31 cells; 14.2%) or expanded clonotypes (187 cells; 85.8%). **(G)** Proportion of cells expressing expanded TCR clonotypes sorted as CD27^{hi} (5 cells; 2.6%) or CD27$^{lo/neg}$ (187 cells; 97.4%); or expressing singlet TCRs sorted as CD27^{hi} (224 cells; 87.8%) or CD27$^{lo/neg}$ (31 cells; 12.2%). **(H)** Proportion of cells expressing expanded TCR clonotypes assigned to cluster 1 (9 cells; 4.7%) or cluster 2 (183 cells; 95.3%); or singlet TCRs assigned to cluster 1 (196 cells; 76.9%) or cluster 2 (59 cells; 23.1%)**. (I-J)** The most abundant clones from each donor displayed very few differences from one another. However, these clones did show distinct Vγ gene segment usage **(I)**, which was validated by their prevalence in previous NGS TCR repertoire analysis of these donors (Davey et al., 2017) **(J)**. Clone A also expressed a rearranged TCRβ gene (*TRBV11-3*). Clone K was from a female donor and expressed *XIST*, a gene associated with X chromosome inactivation.

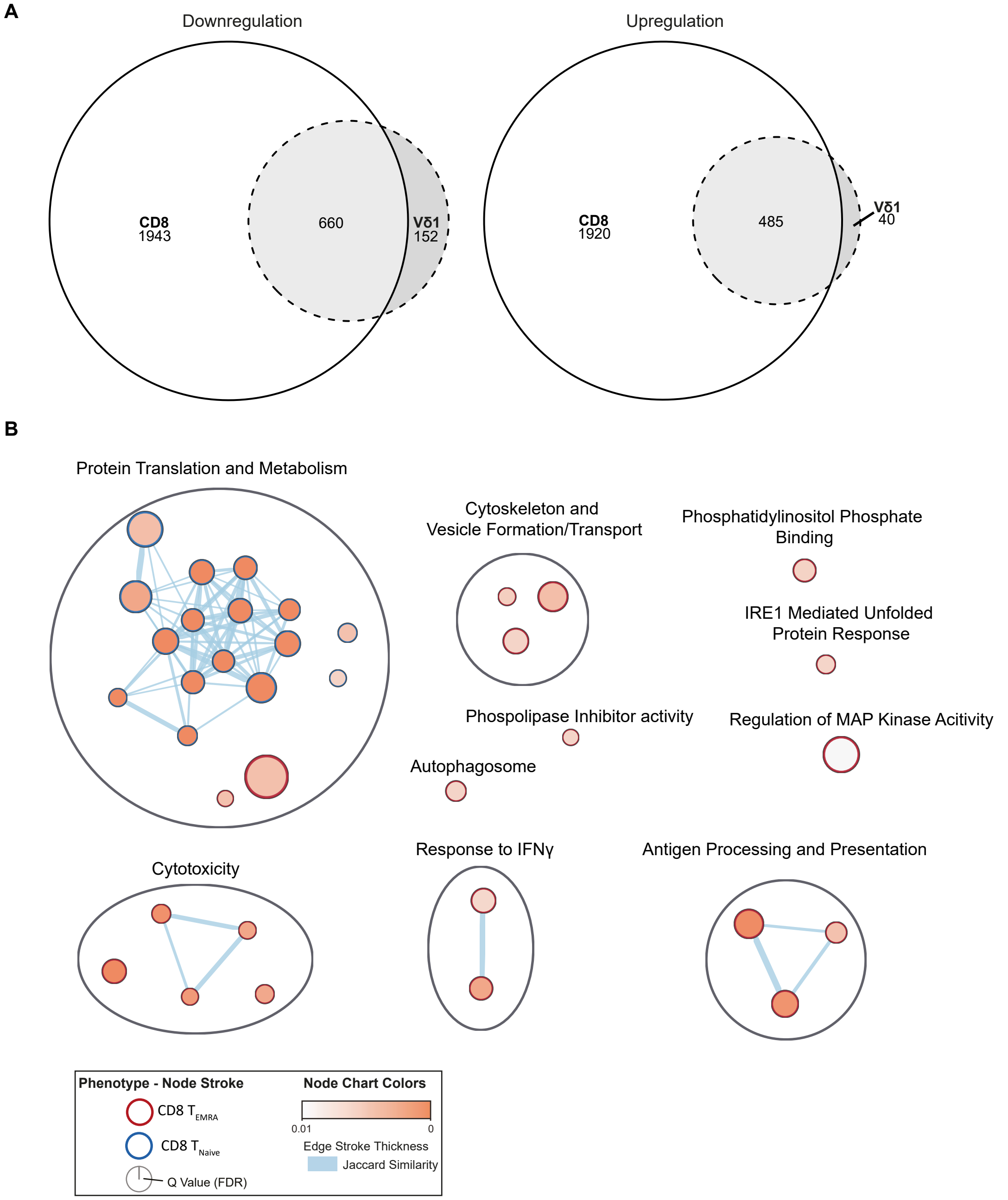
A
Downregulation
CD8
1943
660
Vδ1
152
Upregulation
CD8
1920
485
Vδ1
40
B
Protein Translation and Metabolism
Cytoskeleton and
Vesicle Formation/Transport
Phosphatidylinositol Phosphate
Binding
IRE1 Mediated Unfolded
Protein Response
Phospolipase Inhibitor activity
Regulation of MAP Kinase Acitivity
Autophagosome
Cytotoxicity
Response to IFNγ
Antigen Processing and Presentation
Phenotype - Node Stroke
CD8 T EMRA
CD8 T Naive
Q Value (FDR)
Node Chart Colors
0.01
0
Edge Stroke Thickness
Jaccard Similarity

Figure S2

Figure S2. Bulk RNA sequencing on sorted Vδ1 populations reveals transcriptomic similarity between Vδ1 and CD8 populations. Related to Figure 2.

(A) Differential gene expression between Vδ1 and CD8 T-cell populations revealed a number of shared differentially expressed transcripts. These were delineated into subsets of genes which downregulated in (left) or upregulated (right) upon differentiation from $T_{naïve}$ to $T_{effector}/T_{EMRA}$ cells.

(B) Geneset enrichment analysis (GSEA) using the GO database revealed enrichment of 12 pathways associated with protein translation in the CD8 $T_{naïve}$ population. In contrast, pathways associated with Cytotoxicity, Cellular response to IFNγ, and cellular metabolism were enriched in CD8 T_{EMRA} cells. Genes differentially expressed between CD8 populations were used as input. Cytoscape was used to produce the enrichment map, where the size of the node indicates the size of the geneset, edge thickness represents Jaccard similarity scores between the gene sets, and node colour is based on FDR value. Only genesets with an FDR below 0.001 are shown.

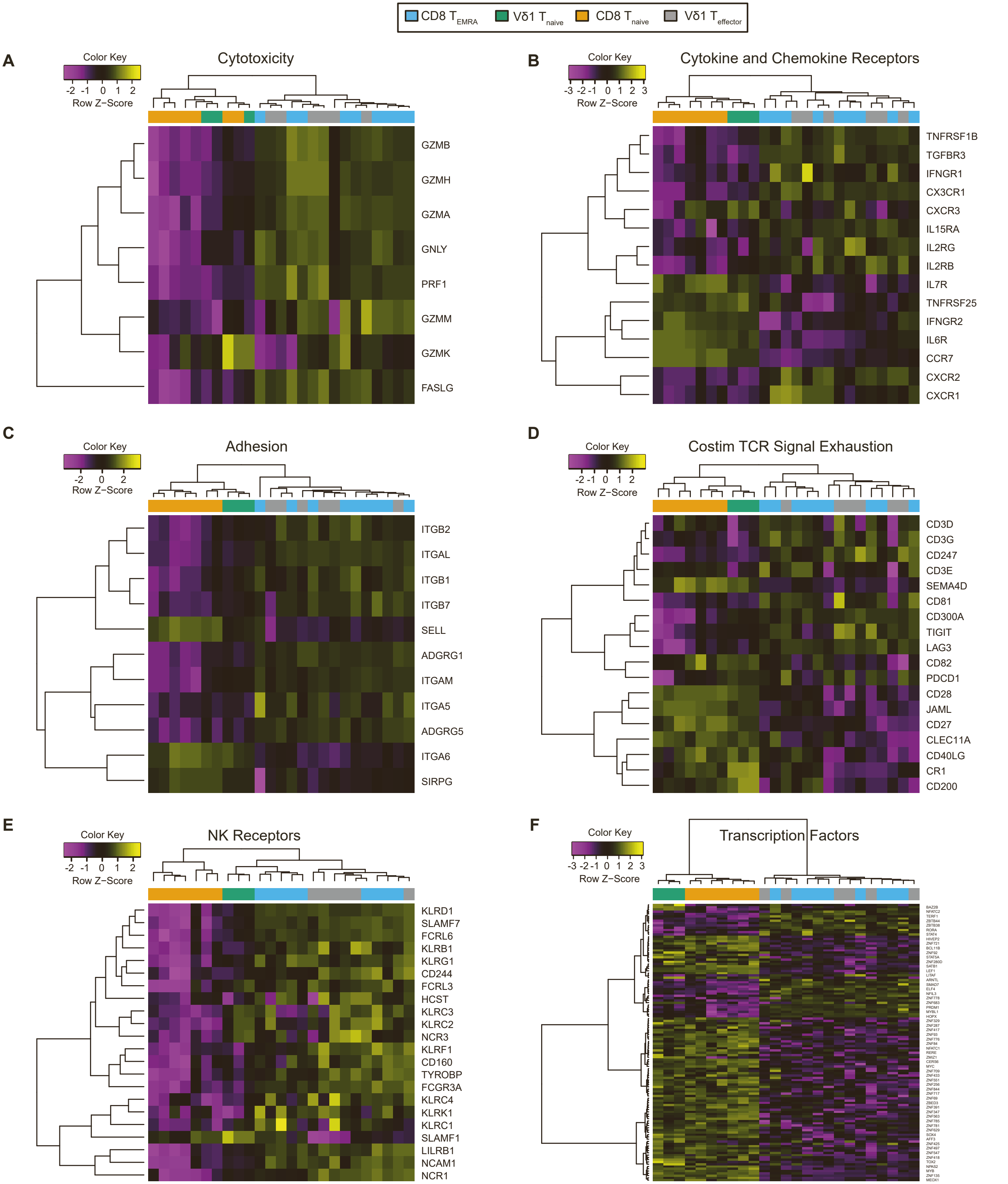
CD8 T_EMRA
Vδ1 T_naive
CD8 T_naive
Vδ1 T_effector
A
Color Key
-2 -1 0 1 2
Row Z−Score
Cytotoxicity
GZMB
GZMH
GZMA
GNLY
PRF1
GZMM
GZMK
FASLG
B
Color Key
-3 -2 -1 0 1 2 3
Row Z−Score
Cytokine and Chemokine Receptors
TNFRSF1B
TGFBR3
IFNGR1
CX3CR1
CXCR3
IL15RA
IL2RG
IL2RB
IL7R
TNFRSF25
IFNGR2
IL6R
CCR7
CXCR2
CXCR1
C
Color Key
-2 0 2
Row Z−Score
Adhesion
ITGB2
ITGAL
ITGB1
ITGB7
SELL
ADGRG1
ITGAM
ITGA5
ADGRG5
ITGA6
SIRPG
D
Color Key
-2 -1 0 1 2
Row Z−Score
Costim TCR Signal Exhaustion
CD3D
CD3G
CD247
CD3E
SEMA4D
CD81
CD300A
TIGIT
LAG3
CD82
PDCD1
CD28
JAML
CD27
CLEC11A
CD40LG
CR1
CD200
E
Color Key
-2 -1 0 1 2
Row Z−Score
NK Receptors
KLRD1
SLAMF7
FCRL6
KLRB1
KLRG1
CD244
FCRL3
HCST
KLRC3
KLRC2
NCR3
KLRF1
CD160
TYROBP
FCGR3A
KLRC4
KLRK1
KLRC1
SLAMF1
LILRB1
NCAM1
NCR1
F
Color Key
-3 -2 -1 0 1 2 3
Row Z−Score
Transcription Factors

Figure S3

Figure S3. Bespoke gene lists show close concordance between Vδ1 $T_{effector}$ and CD8 T_{EMRA} cells. Related to Figure 3.

(A-E) Heatmaps of bespoke gene lists linked to cytotoxicity, cytokine and chemokine receptors, adhesion, costimulation and TCR signalling and exhaustion, and NK receptors were created, utilising hierarchical clustering algorithms. In all instances, Vδ1 $T_{effector}$ clustered with CD8 T_{EMRA}, whereas Vδ1 $T_{naïve}$ clustered with CD8 $T_{naïve}$.

(F) 122 transcription factors differentially expressed between Vδ1 $T_{naïve}$ and $T_{effector}$ cells were plotted as a heatmap utilising hierarchical clustering.

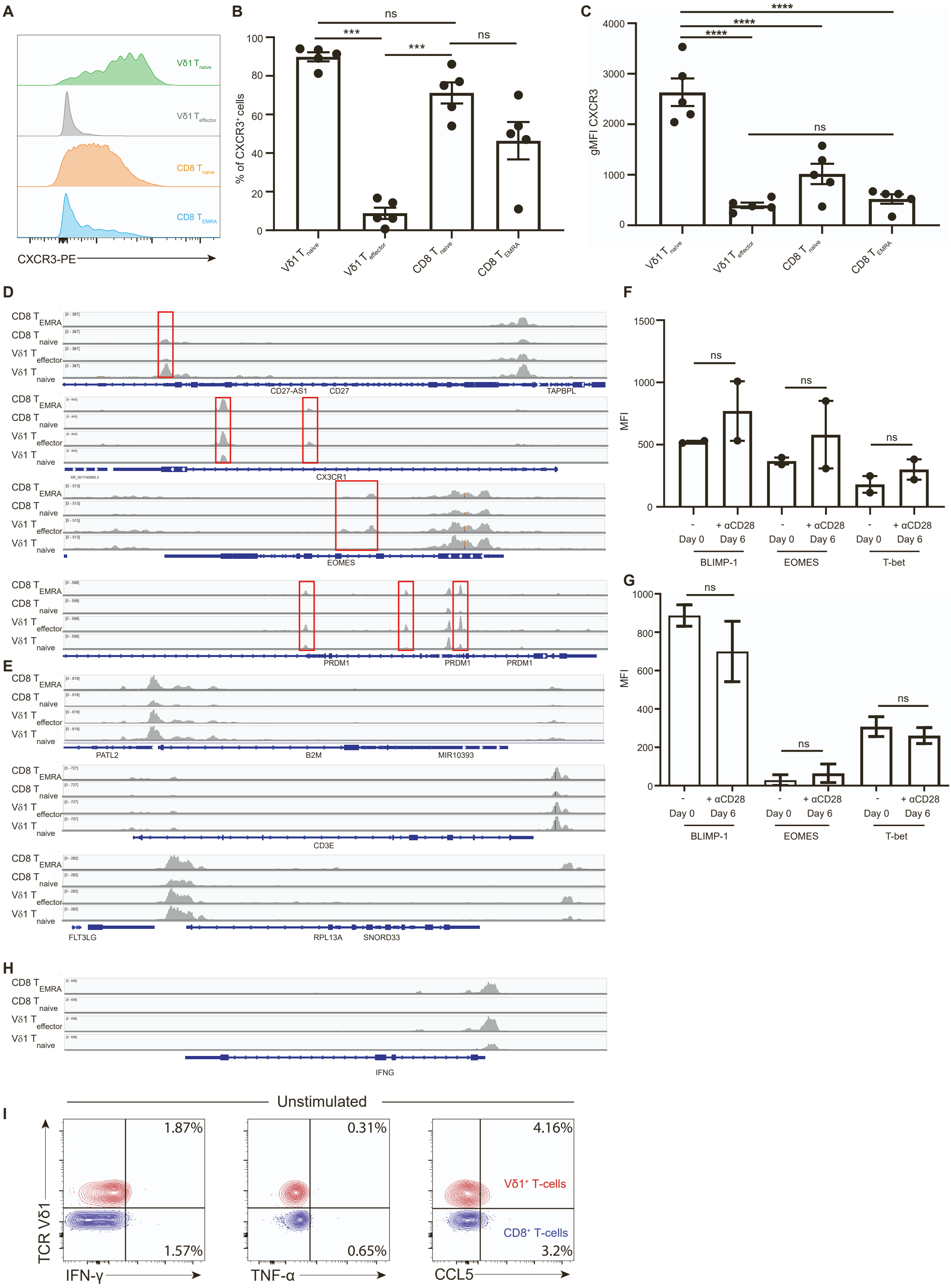

A
Vδ1 T naive
Vδ1 T effector
CD8 T naive
CD8 T EMRA
CXCR3-PE
B
% of CXCR3⁺ cells
ns
***
***
ns
100
80
60
40
20
0
Vδ1 T naive
Vδ1 T effector
CD8 T naive
CD8 T EMRA
C
gMFI CXCR3
****
****
****
ns
4000
3000
2000
1000
0
Vδ1 T naive
Vδ1 T effector
CD8 T naive
CD8 T EMRA
D
CD8 T EMRA
CD8 T naive
Vδ1 T effector
Vδ1 T naive
CD27-AS1
CD27
TAPBPL
XR_001740660.2
CX3CR1
EOMES
PRDM1
PRDM1
PRDM1
E
PATL2
B2M
MIR10393
CD3E
FLT3LG
RPL13A
SNORD33
F
MFI
1500
1000
500
0
ns
ns
ns
-
Day 0
+ αCD28
Day 6
BLIMP-1
EOMES
T-bet
G
MFI
1000
800
600
400
200
0
ns
ns
ns
-
Day 0
+ αCD28
Day 6
BLIMP-1
EOMES
T-bet
H
CD8 T EMRA
CD8 T naive
Vδ1 T effector
Vδ1 T naive
IFNG
I
Unstimulated
TCR Vδ1
1.87%
1.57%
IFN-γ
0.31%
0.65%
TNF-α
4.16%
3.2%
CCL5
Vδ1⁺ T-cells
CD8⁺ T-cells

Figure S4

Figure S4. Flow cytometry and ATAC comparison of Vδ1 and CD8 T cell subsets. Related to Figures 3 and 4.

(A-C) Expression of CXCR3 on Vδ1 and CD8 T-cell subsets. Prevalence of Vδ1 and CD8 T-cell subsets expressing CXCR3 was determined using flow cytometry. **(A)** Representative histograms for flow cytometric analysis. Five donors were collated, and bar charts representing % of CXCR3 positive cells **(B)** and CXCR3 gMFI **(C)** are shown. Data were compared with one-way ANOVA with Tukey's multiple comparisons (P < 0.001; ***).

(D) ATAC sequencing peaks from cell populations were visualised using IGV Genome viewer from a single donor. Peaks are shown for the genes: *CD27*, *CX3CR1*, *EOMES*, and *PRDM1* and highlight differences between effector and naïve populations. Differing peaks of interest between the cell populations are highlighted in the red boxes. **(E)** No differences between peak size were seen between the populations in terms of *CD3E* and the housekeeping genes *B2M* and *RPL13A*.

(F-G) Minimal and insignificant effects of CD28 ligation on expression of transcription factors in enriched Vδ1 $T_{\text{naïve}}$ **(F)** and CD8 $T_{\text{naïve}}$ **(G)** subsets. Flow cytometric analysis of BLIMP-1, Eomes and T-bet expression in $T_{\text{naïve}}$ cells on day 0 (unstimulated) and day 6 of stimulation with plate-bound anti-CD28 antibodies. The bar charts represents data from two donors. Data were analysed by one-way ANOVA with Tukey's multiple comparisons.

(H) ATAC sequencing peaks from cell populations were visualised using IGV Genome viewer from a single donor. Peaks are shown for *IFNG* and differences are seen between T_{effector}/T_{EMRA} and $T_{\text{naïve}}$ populations.

(I) Cytokine production by unstimulated Vδ1 and CD8 T-cells. Overlaid contour flow cytometry plots of gated populations of Vδ1^{+} T-cells (top) and CD8^{+} T-cells (bottom).

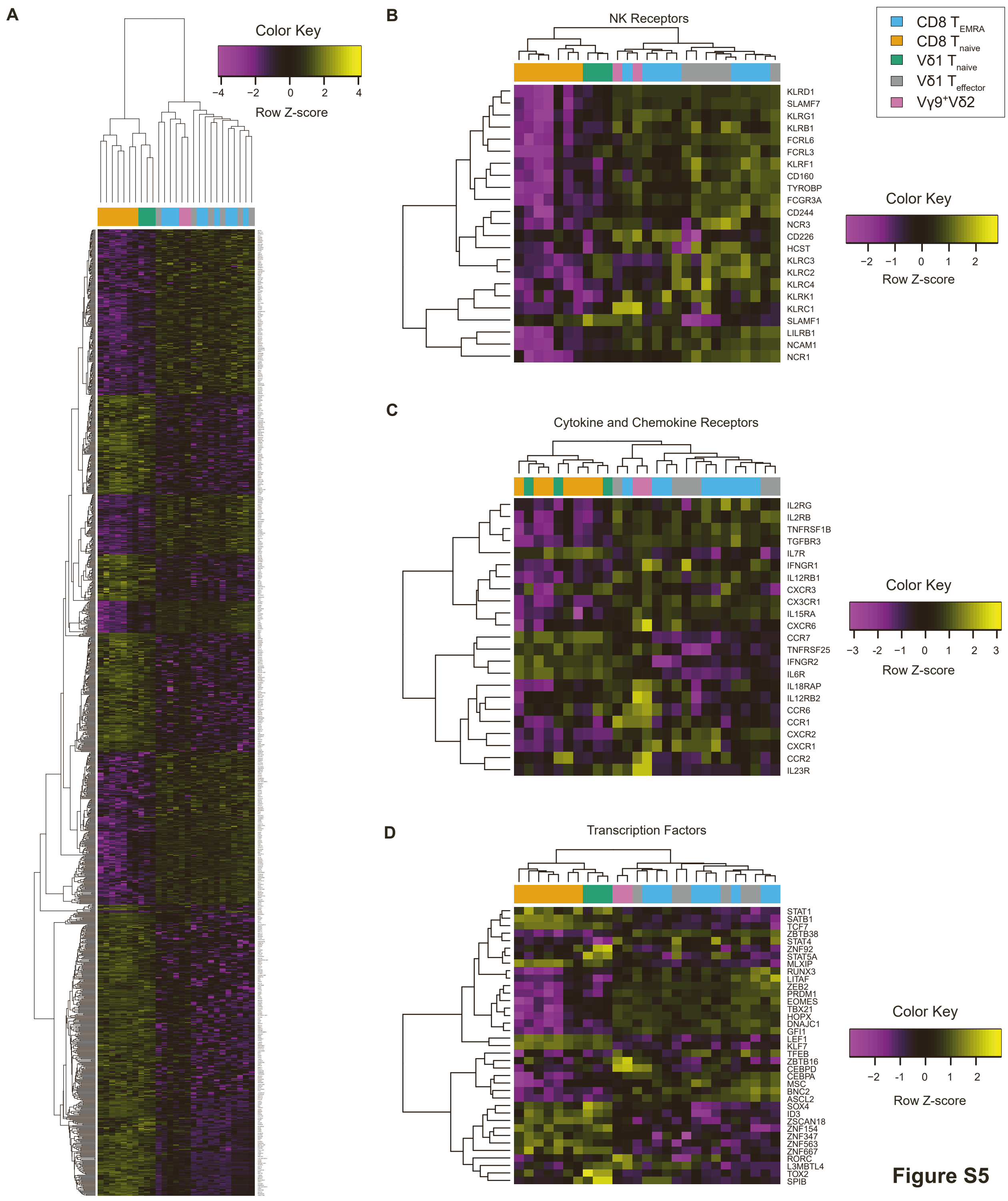
A
Color Key
−4 −2 0 2 4
Row Z-score
B
NK Receptors
CD8 T_EMRA
CD8 T_naive
Vδ1 T_naive
Vδ1 T_effector
Vγ9+Vδ2
KLRD1
SLAMF7
KLRG1
KLRB1
FCRL6
FCRL3
KLRF1
CD160
TYROBP
FCGR3A
CD244
NCR3
CD226
HCST
KLRC3
KLRC2
KLRC4
KLRK1
KLRC1
SLAMF1
LILRB1
NCAM1
NCR1
Color Key
−2 −1 0 1 2
Row Z-score
C
Cytokine and Chemokine Receptors
IL2RG
IL2RB
TNFRSF1B
TGFBR3
IL7R
IFNGR1
IL12RB1
CXCR3
CX3CR1
IL15RA
CXCR6
CCR7
TNFRSF25
IFNGR2
IL6R
IL18RAP
IL12RB2
CCR6
CCR1
CXCR2
CXCR1
CCR2
IL23R
Color Key
−3 -2 −1 0 1 2 3
Row Z-score
D
Transcription Factors
STAT1
SATB1
TCF7
ZBTB38
STAT4
ZNF92
STAT5A
MLXIP
RUNX3
LITAF
ZEB2
PRDM1
EOMES
TBX21
HOPX
DNAJC1
GFI1
LEF1
KLF7
TFEB
ZBTB16
CEBPD
CEBPA
MSC
BNC2
ASCL2
SOX4
ID3
ZSCAN18
ZNF154
ZNF347
ZNF563
ZNF667
RORC
L3MBTL4
TOX2
SPIB
Color Key
−2 -1 0 1 2
Row Z-score

Figure S5

Figure S5. Transcriptomic analysis of Vγ9⁺Vδ2⁺ cells reveals an effector-like program. Related to Figure 5.

(A) Hierarchically clustered heatmap of the core 1150 transcripts differentially expressed in both Vδ1 and CD8 T-cell comparisons highlighted similarity between Vδ1 $T_{effector}$ cells, CD8 T_{EMRA} cells, and $V\gamma9^{+}V\delta2^{+}$ cells. Genes are plotted row-wise and scaled to represent Z-scores across samples.

(B-C) Heatmaps of bespoke gene lists linked to NK receptors, and cytokine and chemokine receptors were created, utilising hierarchical clustering algorithms.

(D) A heatmap of transcription factors expression in Vδ1, CD8, and $V\gamma9^{+}V\delta2^{+}$ T-cell populations was plotted utilising hierarchical clustering. This analysis includes a broader range of transcription factors than Figure S3F incorporating those differentially expressed between Vδ1 and CD8 T-cell populations, and those enriched in the $V\gamma9^{+}V\delta2^{+}$ compartment (*RORC*, *PLZF*, *SATB1*, and *CEBPD*).

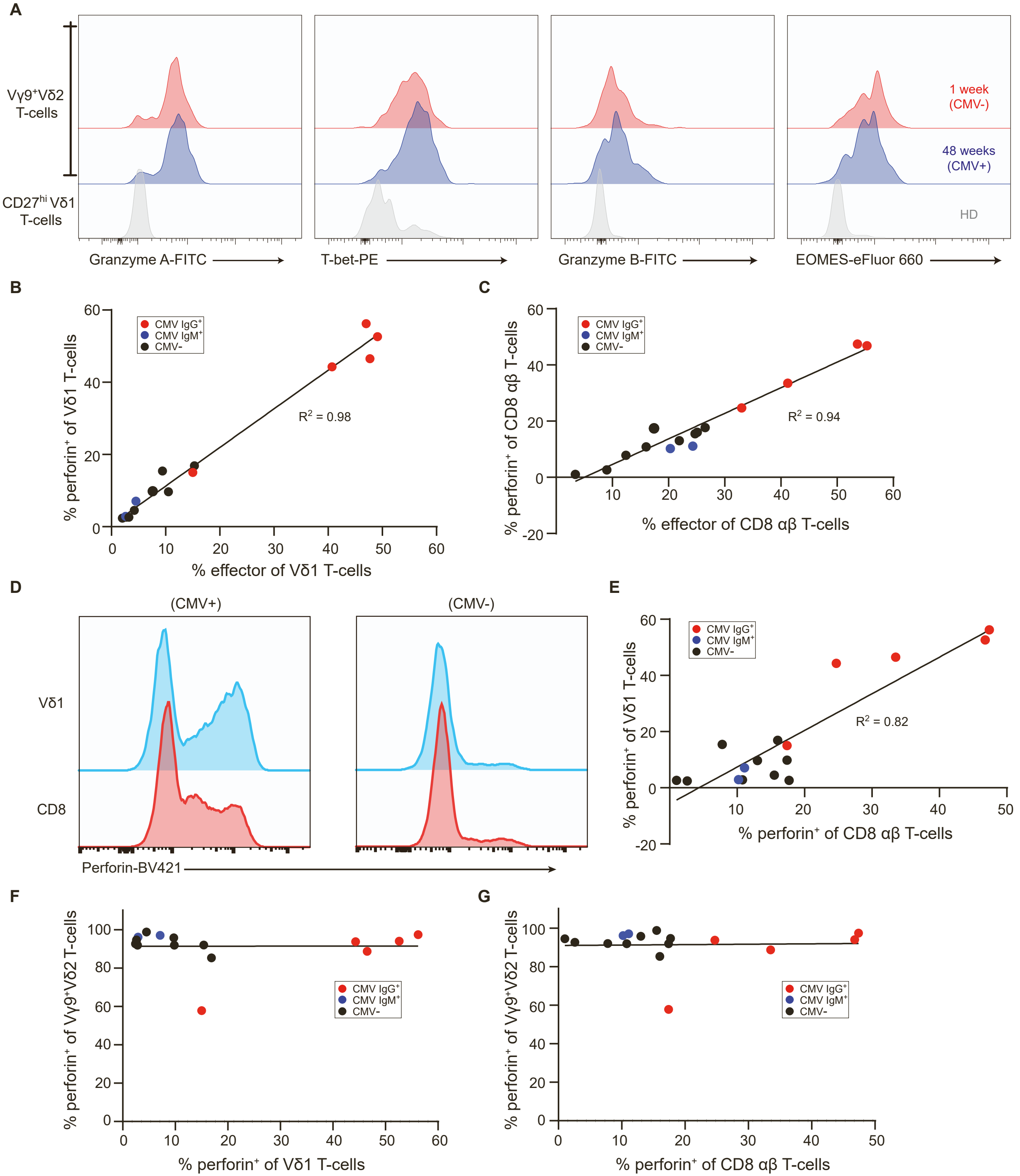

A
Vγ9⁺Vδ2 T-cells
CD27hi Vδ1 T-cells
1 week (CMV-)
48 weeks (CMV+)
HD
Granzyme A-FITC
T-bet-PE
Granzyme B-FITC
EOMES-eFluor 660
B
CMV IgG⁺
CMV IgM⁺
CMV-
R² = 0.98
% perforin⁺ of Vδ1 T-cells
% effector of Vδ1 T-cells
C
CMV IgG⁺
CMV IgM⁺
CMV-
R² = 0.94
% perforin⁺ of CD8 αβ T-cells
% effector of CD8 αβ T-cells
D
(CMV+)
(CMV-)
Vδ1
CD8
Perforin-BV421
E
CMV IgG⁺
CMV IgM⁺
CMV-
R² = 0.82
% perforin⁺ of Vδ1 T-cells
% perforin⁺ of CD8 αβ T-cells
F
CMV IgG⁺
CMV IgM⁺
CMV-
% perforin⁺ of Vγ9⁺Vδ2 T-cells
% perforin⁺ of Vδ1 T-cells
G
CMV IgG⁺
CMV IgM⁺
CMV-
% perforin⁺ of Vγ9⁺Vδ2 T-cells
% perforin⁺ of CD8 αβ T-cells

Figure S6

Figure S6. Adaptive differentiation of human γδ T-cells *in vivo*. Related to Figure 6.

(A) Vγ9$^+$Vδ2$^+$ T-cells remain positive for Granzyme A, T-bet, Granzyme B, and Eomes even after CMV seroconversion.

(B) Correlation of percentage perforin and CD27$^{lo/neg}$ status in the Vδ1 compartment in healthy children (R^2 = 0.98, Spearman coefficient = 0.95).

(C) Correlation of percentage perforin and $T_{effector}$ (T_{CM} + T_{EM} + T_{EMRA}) status in the CD8 T-cell compartment in healthy children (R^2 = 0.94, Spearman coefficient = 0.88).

(D) Perforin staining in total Vδ1$^+$ T-cells versus total CD8 T-cells in a representative CMV$^+$ healthy donor (HV16, age 78 months), and a CMV^{neg} healthy donor (HV18, age 15 months).

(E) Percentage of perforin$^+$ cells in total Vδ1$^+$ T-cells cells correlates with perforin staining in CD8 T-cells (R^2 = 0.82, Spearman coefficient = 0.74).

(F) Percentage of perforin$^+$ cells in total Vγ9$^+$Vδ2$^+$ cells does not correlate with perforin staining in Vδ1$^+$ T-cells.

(G) Percentage of perforin$^+$ cells in total Vγ9$^+$Vδ2$^+$ cells does not correlate with perforin staining in CD8$^+$ T-cells.
